# BBB-Permeable PROTACs: Where Do We Stand?

**DOI:** 10.1021/acsmedchemlett.5c00768

**Published:** 2026-03-05

**Authors:** Serena Francisco, Giulia Apprato, Matteo Rossi Sebastiano, Giuseppe Ermondi, Giulia Caron

**Affiliations:** Department of Molecular Biotechnology and Health Sciences, MedChemBeyond Lab, University of Torino, Piazza Nizza 44bis, 10126 Torino, Italy

**Keywords:** PROTACs, Blood−Brain Barrier, Central Nervous System, Permeability, bRo5

## Abstract

The development of CNS-active PROTACs is limited by the blood–brain barrier (BBB). This Microperspective briefly reviews BBB physiology and critically evaluates current *in silico*, *in vitro*, and *in vivo* models used to assess brain penetration. These considerations enable a unified analysis of the 30 published 30 CNS-targeted degraders compiled here for the first time, highlighting the lack of a consistent design strategy and offering perspectives for future programs.

## Graphical abstract

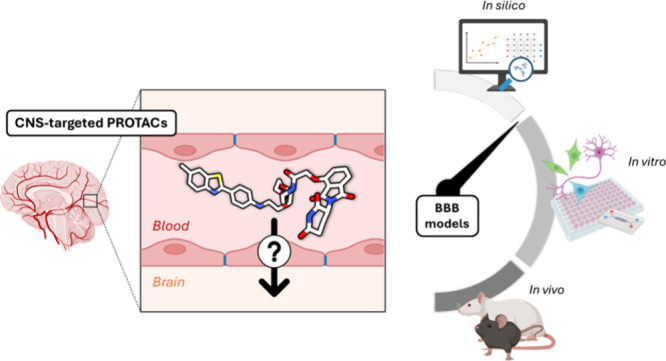

As the population ages, the incidence of neurodegenerative disorders (NDDs)—including Alzheimer’s disease (AD), Parkinson disease (PD), frontotemporal dementia (FTD), Huntington disease (HD), and amyotrophic lateral sclerosis (ALS)—continues to rise, while therapies still largely rely upon symptom relief rather than targeting primary disease causes.^1^ Drug discovery in neurological disorders lags behind other fields due to several key challenges: (a) a limited understanding of the underlying cellular mechanisms, which restricts the number of validated targets, (b) the prevalence of targets that are difficult to modulate with molecules adhering to the Lipinski’s Rule of Five (Ro5),^2^ which are not well suited for protein–protein interactions (PPIs),^3^ and (c) the intrinsic difficulty faced by compounds in crossing the blood–brain barrier (BBB).

PROteolysis TArgeting Chimeras (PROTACs) are heterobifunctional molecules that induce the degradation of a target protein through the ubiquitin-proteasome system (UPS). Specifically, PROTACs consist of a warhead, a linker, and an E3 ligand (). While the warhead engages in interactions with the protein of interest (POI), the E3 ligand binds to the E3 ligase. The resulting assembly is called the ternary complex (TC). In this way, a PROTAC molecule is able to bring the E3 ligase and the POI into the proximity to induce ubiquitination and degradation of the latter. PROTACs feature an “event-driven” mode of action (MoA) and eliminate the protein via targeted protein degradation (TPD) already at substoichiometric concentrations.^4^

In the neurological landscape, PROTACs may offer an alternative strategy by inducing the degradation of target proteins involved in NDD and/or their aggregates (Table 1), relying on a stable TC rather than high warhead–protein affinity.^5^ However, the physicochemical profile of PROTACs places them in beyond Rule-of-Five (bRo5) space. Even though specific considerations have been made for non-PROTAC bRo5 compounds (e.g., macrocycles),^6,7^ this chemical space typically remains unfavorable for efficient BBB penetration.^8^

**Table 1:** Common Molecular Targets of NDDs Grouped into Proteins Giving Rise to Aggregates,^9^ Protein Kinases,^10,11^ Receptors (LDR1, RAGE),^12^ and Catalytic Subunits from Larger Complexes (PSEN1, PSEN2)

Protein category	Protein name (Gene name)	UniProt ID
Aggregate-forming	Tau (*MAPT*)	P10636
Aggregate-forming	Amyloid-beta precursor protein (*APP*)	P05067
Aggregate-forming	Alpha-synuclein (*SNCA*)	P37840
Aggregate-forming	TDP-43 (*TARDBP*)	Q13148
Kinases	GSK-3 beta (*GSK3B*)	P49841
Kinases	CDK5 (*CDK5*)	Q00535
Kinases	LRRK2 (*LRRK2*)	Q5S007
Receptors	RAGE (*AGER*)	Q15109
Lipid transport	Apolipoprotein E (*APOE*)	P02649
Catalytic subunits	Presenilin-1 (*PSEN1*)	P49768
Catalytic subunits	Presenilin-2 (*PSEN2*)	P49810

Despite these challenges, PROTACs crossing the BBB to modulate targets in the central nervous system (CNS) have been discovered (these are discussed later on, while related references can be found in ). For instance, Holmqvist and colleagues developed an innovative platform enabling the simultaneous synthesis and testing of bifunctional degraders by varying warheads, linkers, and E3 ligands.^13^ This orthogonally reactive linker system ultimately yielded the GSK3 degrader KH1, showing favorable kinetics and BBB permeability in mouse models. Although this platform represents a major advance in CNS-oriented PROTAC discovery, the related *in vitro* validation relies on simplified cell systems (e.g., HEK293 and SH-SY5Y cells) in both early screening and follow-up validation and, crucially, these *in vitro* assays do not account for BBB permeability. This example highlights the need to comprehensively evaluate all BBB-permeable PROTACs and assess the approaches used to demonstrate their permeability. Therefore, in this work, we (a) briefly summarize key aspects of BBB physiology and pathology of potential relevance for the medicinal chemistry community, (b) critically review the computational and experimental systems currently used across drug discovery stages to assess brain penetration, and (c) analyze published PROTACs reported to act on CNS targets with the aim to deconvolute the strategies employed for verifying their ability to cross the BBB.

## Physiology, Aging and Pathology of the BBB

The BBB consists of brain endothelial cells (BECs) and separates the brain parenchyma from the bloodstream. This warrants the CNS homeostasis, which is paramount because of the limited regenerative capacity of neural cells.^14^ BECs form the neurovascular unit (NVU) together with pericytes, astrocytes, microglia, neurons, smooth muscle cells and the basement membrane (Figure 1A).^15^ These components exert diverse functions and altogether account for the complexity of this barrier.

**Figure 1:**
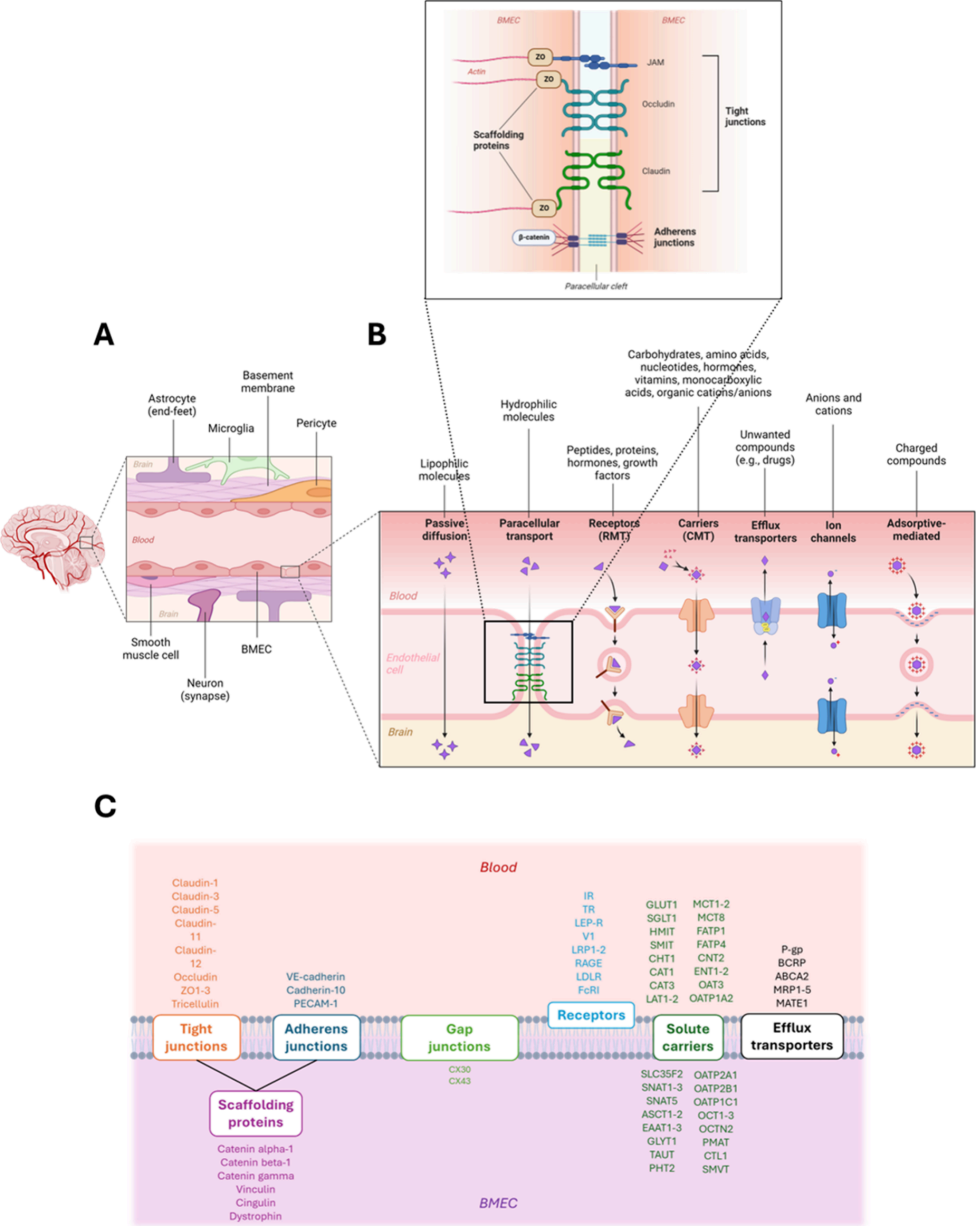
(A) The NVU and its components. (B) Junctional molecules sealing together BECs (top) and transport routes at the BBB (bottom). (C) Main examples of targets for different protein categories found at the membrane sites of BECs.

Although the BBB shares functional similarities with the intestinal barrier (IB), it is substantially more restrictive with respect to passive molecular permeation. This is largely due to differences in junctional protein expression and surrounding cells.^16^ Moreover, while the IB consists of epithelial cells, the BBB is made of endothelial cells and displays heterogeneous composition across different brain areas, thus posing additional challenges to therapy and drug targeting.^17^

BECs feature a proper signature encompassing a characteristic expression of tight junctions and active influx/efflux transporters, lack of fenestrations, high mitochondrial content, peculiar membrane composition and scarcity of transcytotic vesicles.^18–20^ Pericytes and astrocytes regulate cerebral blood flow along with smooth muscle cells, and support barrier integrity.^18,21,22^ Microglia are resident brain immune cells that clear the cerebral microenvironment from debris and toxic substances.^23^ The basement membrane is the acellular component of the NVU providing an anchoring platform for cellular components.^14^ Finally, a key factor influencing BECs’ gene expression is represented by shear stress exerted on BECs and generated by the blood flow.^24^

As the forming units of both a physical and a functional barrier, BECs express a range of membrane proteins that maintain barrier integrity and regulate substance exchange. They are sealed together by tight junctions (claudins—especially claudin-5—and occludin), adherens junctions (mainly VE-cadherin), and junctional adhesion molecules (JAM1–3) (Figure 1B,C).^25^ These components strongly limit paracellular diffusion at the BBB and account for high transendothelial electrical resistance (TEER) values. They also polarize BECs into apical and basolateral regions. Scaffolding proteins anchor these junctional complexes to the cytoskeleton and are mainly represented by zonula occludens 1 to 3 (ZO1–3).^14^ Transport of solutes across BECs occurs via carrier-mediated transport (CMT, gradient-driven transport performed by solute carriers like GLUT1, LAT1, and CAT1/3),^26^ receptor-mediated transport/transcytosis (RMT, for larger ligands like transferrin and insulin), adsorptive-mediated transcytosis (receptor-free, charge-driven event at specific membrane sites)^27^ and efflux transporters (active extrusion of substances into the bloodstream mediated by transporters such as P-gp, BCRP, and MRPs) (Figure 1B,C).^14^ Additional proteins worth mention include the lipid transporter Mfsd2a, which mediates docosahexaenoic acid (DHA) uptake and suppresses vesicular trafficking via inhibition of caveolae-mediated transcytosis—thus accounting for the restrictive properties of BECs compared to peripheral endothelial cells.^20^ Finally, gap junction proteins (e.g., CX30, CX43) facilitate BECs’ intercellular communication. More details about key membrane components of BEC are available in .

Aging leads to progressive BBB breakdown via oxidative stress, inflammation, and structural deterioration of BECs and NVU components (Figure 2).^28^ Such alterations are amplified in neurodegenerative disorders such as AD and PD, exposing the brain to blood-borne substances, including potentially neurotoxic compounds or therapeutics.^18^

**Figure 2:**
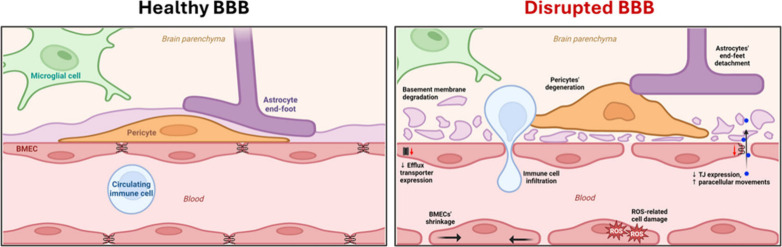
Schematic illustration of the healthy BBB (left) and main events leading to BBB disruption (right panel). These changes include reduced expression of key transporters (P-gp, GLUT1, LRP1), tight junction loss, immune infiltration, astrocyte detachment, and pericyte dropout.^15,28–30^

## Current Models of the BBB and Considerations for PROTAC Drug Discovery

Several medicinal chemistry strategies can improve brain delivery, including increasing passive permeability, reducing active efflux (e.g., via P-gp or BCRP) and, less commonly, exploiting carrier-mediated transcytosis through prodrug design.^31^ However, in practice, efforts mainly focus on boosting passive permeability. This focus is essential because, although BBB integrity might be compromised in NDDs and aging, such disruption is spatially heterogeneous^32^ and insufficient to guarantee uniform drug distribution. This means that relying on disease- and age-driven leakage might not be a clinically valid delivery strategy.

To support optimization efforts, brain-penetration tools are used throughout the discovery pipeline: *in silico* models guide early design, *in vitro* assays inform lead optimization, and *in vivo* studies provide the most reliable measure of brain exposure for advanced candidates. For CNS-targeted PROTACs, the same general principles apply, but some assessment methods may be insufficient, as discussed below.

### *In Silico* Models

Common medicinal chemistry strategies to enhance CNS penetration of drug candidates include the modulation of molecular properties: (a) increasing lipophilicity (clogP/clogD), (b) reducing the hydrogen bond donor count (nHDon or HBD), (c) lowering polarity (TPSA), (d) enhancing molecular rigidity, and (e) decreasing ionization (p*K*_a_).^31^ These descriptors can be used alone or in combination (e.g., rule-based models). Quantitative structure–property relationship (QSPR) models have also been described (their review is beyond the aim of this paper).

However, when considering bRo5 derivatives, any model for predicting BBB passage faces two main limitations: the scarcity and questionable reliability of experimental data and the uncertainty regarding the identification of the most relevant molecular descriptors. To this respect, evidence suggests that relying solely on basic 2D descriptors is insufficient. For instance, the Pfizer’s CNS Multi-Parameter Optimization (CNS MPO) tool evaluates six key descriptors (ClogP, ClogD, MW, TPSA, HBD, p*K*_a_) to predict BBB permeability of small molecules. Scores range from 0–6, with ≥4 indicating higher likelihood of CNS penetration (Figure 3).^33^ However, the CNS MPO is often not suitable for bRo5 compounds. As a matter of fact, large compounds are often misclassified as nonpermeable despite evidence of the contrary (e.g., cyclosporin A).^34^

**Figure 3:**
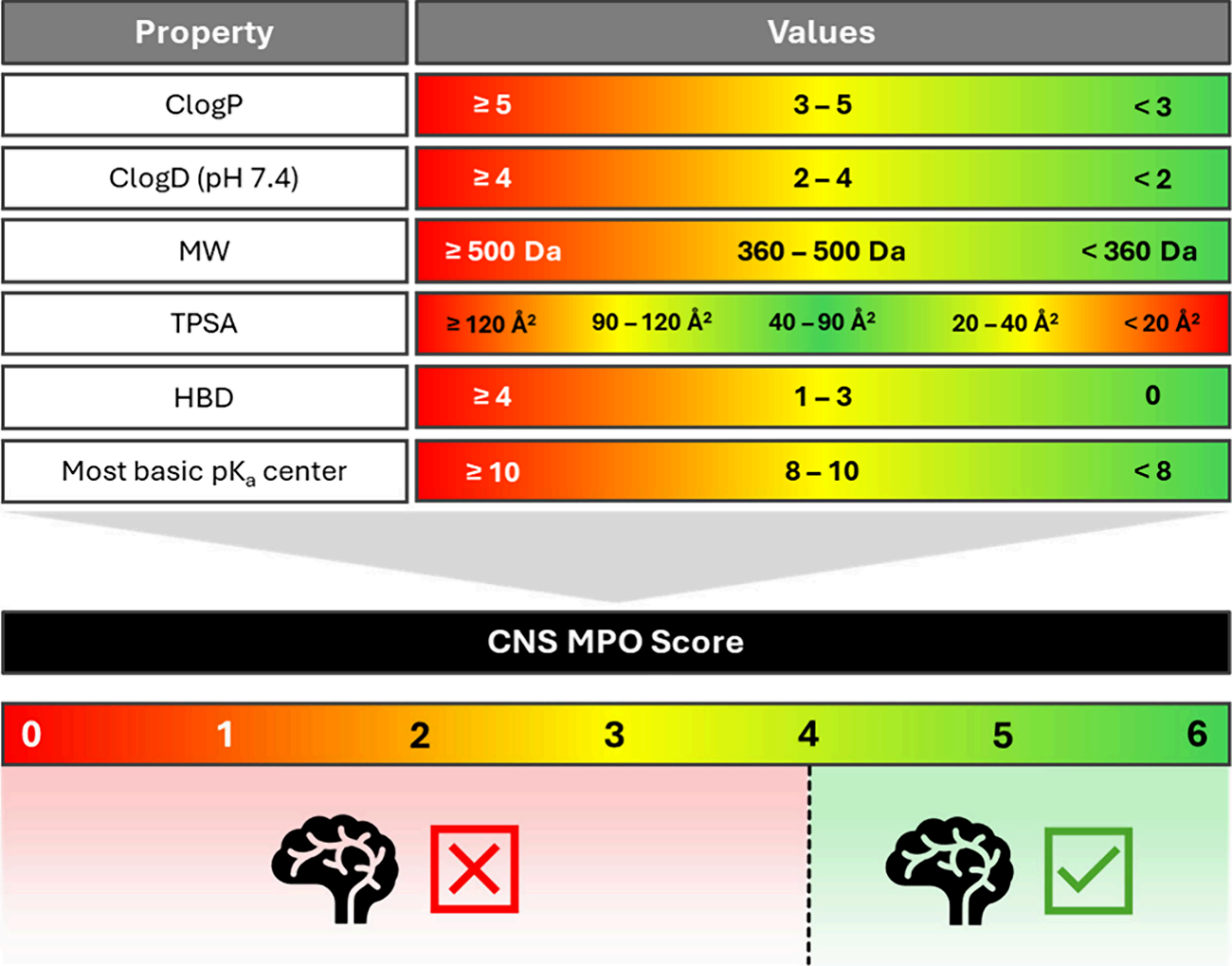
CNS MPO scoring method. ClogP: calculated LogP; ClogD, calculated logD; MW, molecular weight; TPSA: topological polar surface area; HBD: number of hydrogen bond donors.

These shortcomings mostly arise because systems such as the CNS MPO score overlook conformational effects such as the ability of a molecule to form intramolecular hydrogen bonds (IMHBs), which can mask hydrogen bond donors and acceptors from solvent exposure.^35^ Namely, they fail to capture chameleonicity^36^—the capacity of a molecule to adopt different conformations in polar versus nonpolar environments—which plays a crucial role in balancing solubility and membrane permeability.

Mechanistic BBB-permeation models, such as those based on molecular dynamics (MD), are promising but still in an early stage of development and, at present, provide only limited practical value for bRo5 drug discovery.

### *In Vitro* Models

To evaluate BBB permeation *in vitro*, several models have been described and categorized across the literature. Here, we clustered them into noncell-based (or cell-free) and cell-based. The latter are in turn classified into static and dynamic (Figure 4 and ).^37^ Each model offers specific advantages and limitations for the PROTAC drug screening.

**Figure 4:**
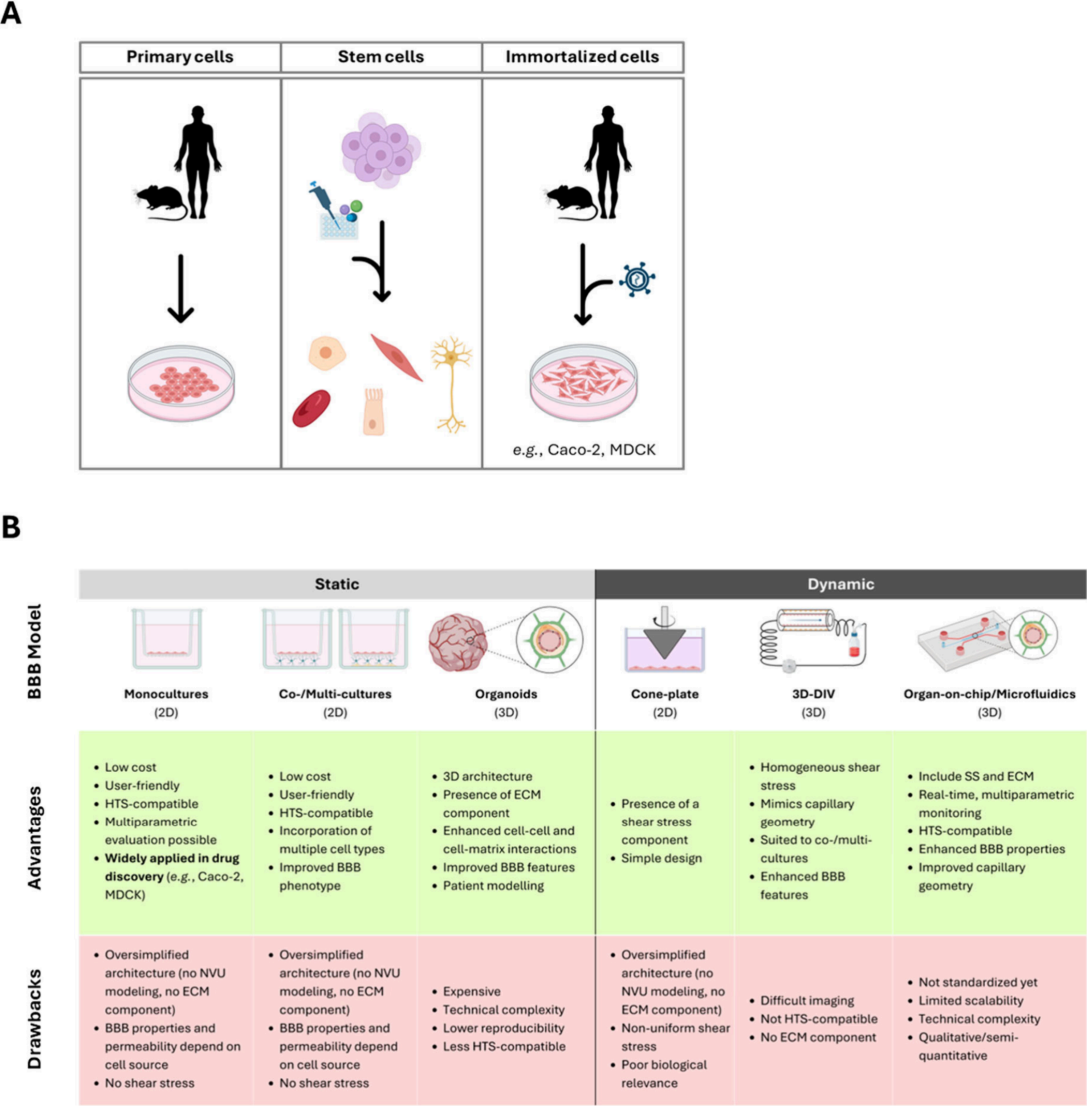
(A) Cell types used for in vitro modeling of the BBB. Primary cells derive from animal models/patients and have short lifespan in culture conditions. Stem cells can be differentiated to different cell lineages by using specific culture conditions and factors. Immortalized lines derive from primary cells properly transformed to significantly extend their lifespan for in vitro studies—though they could also be cancer cells. (B) Summary of main advantages and drawbacks of different in vitro BBB models—which are grouped into static and dynamic. HTS: high-throughput screening; NVU: neurovascular unit; ECM: extracellular matrix; BBB: blood–brain barrier.

Amongst cell-free models, the parallel artificial membrane permeability assay (PAMPA) emerges as a cost-effective method to assess the passive permeability of drug candidates.^38^ The PAMPA-BBB is an optimization of the PAMPA consisting in the use of a mixture of phospholipids specifically tailored for the BBB—typically porcine brain lipid (PBL) dissolved in an organic solvent.^39^ PAMPA systems are well suited for high-throughput screening (HTS) and, thus, are widely used in pharmaceutical settings. However, they do not account for active transport or efflux mechanisms at the BBB. Moreover, PROTACs have been shown to adsorb onto laboratory plastic surfaces, thereby affecting compound recovery and compromising the reliability of the measurements. Overall, PAMPA systems have limited predictive power for PROTACs BBB exposure.^40^

Cell-based systems—which include cells of cerebral and noncerebral origin—employ primary cells (cells isolated from living tissues), stem cells (undifferentiated cells capable of self-renewal and differentiation into specialized cell types), or immortalized (cells that have acquired the ability to proliferate indefinitely in culture) lines (Figure 4A and ). Primary human BECs are closest to the *in vivo* NVU counterparts, though their main drawbacks include limited availability, high cost, and low scalability. On the other hand, nonhuman primary cells come with many species-related differences in gene expression.^37,40,41^ Stem cells—which can be either embryonic, mesenchymal or induced pluripotent (iPSCs)—also display close resemblance to *in vivo* NVU cells upon differentiation and have promising disease modeling potential; however, they rely upon complex reprogramming protocols.^37^ Cerebral immortalized cells are cheaper and with extended lifespan enabling long-term studies, though they lag behind both primary and stem cell-derived NVU components due to their inferior physiological features (e.g., low TEER values, moderate tight junction expression and high paracellular permeability).^41^ Lastly, noncerebral immortalized cells such as Caco-2 and MDCK are still widely used in drug discovery owing to their ease of use for large screenings and suitability for studying efflux mechanisms. They are serving as robust surrogate models for early permeability ranking if the limitations of the model are considered and used within their validation domain.^42^ Figure 4B summarizes the different cell-based systems that are discussed below. These can either be 2D or 3D and are divided into static (no shear stress source: monocultures, co/multicultures, organoids) and dynamic models (shear stress component is mimicked: cone–plate systems, 3D dynamic systems, organ-on-chip/microfluidic devices).^37^

#### Static Systems

2D monolayers are the cheapest and most commonly used *in vitro* models. Drug discovery campaigns make extensive use of the noncerebral cell lines Caco-2 and MDCK-MDR1 for permeability studies, with the latter being particularly well suited to HTS due to their rapid growth and high, stable P-gp expression.^43^ However, since these systems have been designed to study intestinal absorption, their relevance in the context of the CNS and BBB is uncertain. Therefore, *in vitro* systems of cerebral origin may serve as valuable resource. These are mainly Transwell-based monoculture and co/multiculture BBB models that can include one or more NVU cell types. Depending on the organization of cells in the well, co/multicultures can be divided into contact or noncontact models.^44^ These systems are suitable for high-throughput drug screenings and multiparametric evaluations (e.g., TEER measurement, permeability assessment, etc.) but lack physiological accuracy due to their simplicity, as BECs display neither a tubular structure nor proper cell–cell interactions nor a surrounding matrix. On the other hand, organoids—i.e., 3D, self-organizing clusters of cells—offer improved BBB features thanks to their 3D architecture and matrix support, which better recapitulate cell–cell and cell–matrix interactions. Even though they also allow patient-specific modeling, organoids are expensive, less reproducible and scalable, and time-consuming.

#### Dynamic Systems

Among dynamic BBB models, cone–plate models consist of BECs seeded in a plate filled with medium that is displaced by a rotating cone to generate a shear stress-like effect. These systems offer limited uniformity of the resulting medium flow and poor biological relevance. On the other hand, dynamic 3D (3D-DIV) systems incorporate a laminar shear stress source and multiple NVU cells seeded inside hollow fibers, but are poorly scalable and inadequate for imaging and HTS.^37^ Organ-on-chip/microfluidic devices for BBB modeling started being developed in the 2010s and come with different designs.^45^ These systems better mimic the BBB architecture compared with 3D-DIV models thanks to the inclusion of multiple NVU components, including an extracellular matrix. They also allow to mimic pathological states (e.g., AD) and enable real-time, multiparametric analyses.^46^ Contrarily to organoids, these systems require more complex devices, thus, adding a further layer of complexity. Moreover, their reproducibility from one laboratory to another is not trivial, they enable semiquantitative measurements only, and they are poorly scalable and standardized yet.^47–49^ Commercial microfluidic devices are now emerging, which would enhance the scalability potential of these devices for broader applications and HTS.^46^

The quality of an *in vitro*, cell-based BBB model and the permeability of solutes across it are assessed using different strategies. Normally, the integrity of these models is evaluated by measuring the TEER, but also by looking at the paracellular permeability of hydrophilic compounds (sometimes by exploiting fluorescent tags).^50^ Usually, the evaluation of the amount of drug candidate that crosses a given barrier relies upon its detection and quantification in the recipient compartment via LC-MS.^51,52^ This enables us to retrieve the apparent permeability coefficient (*P*_app_). However, when dealing with complex molecules like PROTACs, additional parameters become particularly important—notably, the mass balance (to assess compound recovery and stability) and the quantification of P-gp-mediated efflux.^53^

Human iPSCs represent a promising resource to generate BBB-relevant cells with efflux transporter profiles closer to the human *in vivo* scenario than animal-based models or noncerebral cell lines (e.g., Caco-2, MDCK).^40,54,55^ Recent advances in iPSC maintenance, differentiation, and cryopreservation are making these models increasingly scalable.^40,56^ Encouragingly, permeability measurements using iPSC-derived BECs have shown good correlation with *in vivo* uptake, supporting their future use in high-throughput BBB assays.^55,57,58^ It remains unclear—for PROTAC-related applications—which type of BBB model human iPSCs should be integrated into. As 3D systems more closely mimic the *in vivo* BBB and NVU than 2D models, advances in microfluidic platforms make them strong candidates for future gold-standard BBB models. These systems offer small-scale formats requiring fewer cells and enable cost-effective screening of large compound libraries.^46^

### *In Vivo* Models

Rodents remain a standard in preclinical CNS research.^54,55^ However, they fail to recapitulate the full complexity of human diseases, especially due to species-specific BBB differences in morphology and gene expression.^40,56,59^ Nonhuman primates (NHPs) offer a more similar model to humans compared to rodents, though at the expenses of scalability and ethics.^56^ An additional issue related to the *in vivo* model is the identification of the right parameter to be monitored after administration. Based on the free drug hypothesis (most widely accounted in pharmacokinetics), the passive permeability and availability of a compound in the CNS should be evaluated by measuring the unbound (free) drug concentration in the brain, as only this one is expected to exert an activity at the target site.^57^ The unbound brain-to-plasma ratio *K*_*p,uu*_ (i.e., ratio of the free drug concentration in the brain to the free drug concentration in plasma) tells whether a distribution equilibrium between brain and plasma compartments has been achieved. If this ratio is close to 1, then the free drug concentration in the brain and in plasma is almost the same, and the equilibrium has been reached (ideal scenario). Overall, the assessment of the permeability of compounds in the CNS should be performed by measuring the unbound drug parameters.

## Literature Overview on PROTACs Crossing the BBB

After establishing the structural complexity of the BBB and the methods available for its modeling and compound permeability assessment, we collected published PROTACs targeting CNS proteins. This allowed us to deconvolute the strategies used to verify their BBB penetration efficacy.

A total of 23 studies—published between July 2017 and October 2025—were retrieved, encompassing 30 distinct small-molecule PROTACs. All of the resulting PROTACs are reported together with their target and SMILES codes in . Their bibliographic references are listed in the .

Out of these 30 degraders, three PROTACs have reached clinical evaluation: ARV-102, CFT1946 (NCT05668585), NX-5948 (NCT05131022).^60,61^ Two studies did not have full publications accessible at the time of analysis (ARV-102, NX-5948), making the information reported below fragmentary. The structure of ARV-102, in particular, has not been disclosed yet and herein we reported one available at DC Chemicals,^62^ which is expected to be similar to the actual one. Figure 5A shows selected statistics for the collected *in vitro* and *in vivo* data, while all data can be found in .

**Figure 5:**
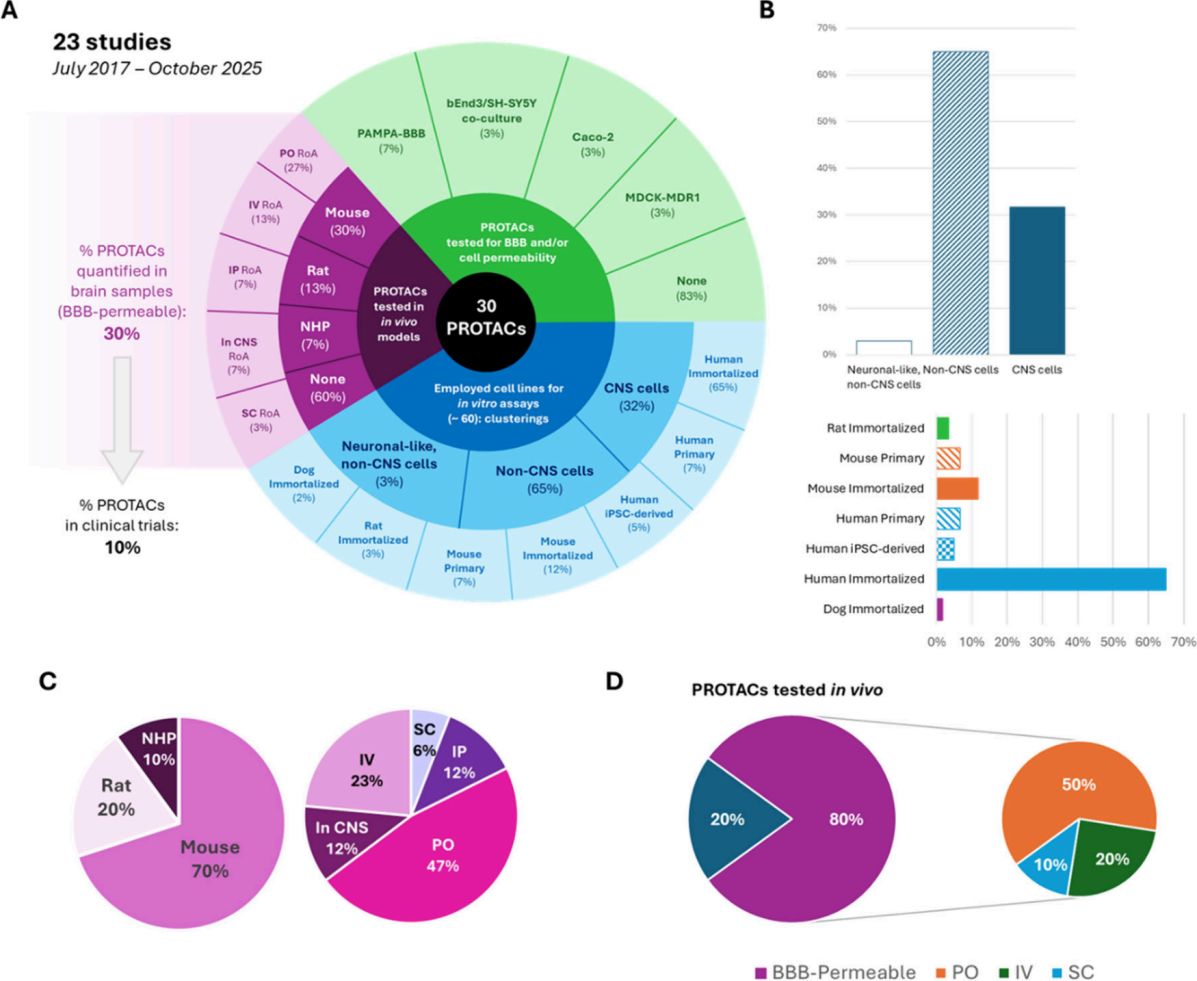
(A) Infographic on collected literature for PROTACs developed either for neurodegeneration and/or with proven BBB permeability. Different percentages of all 30 PROTACs are shown. The amounts of PROTACs tested for either BBB or cell membrane permeability are reported in the slices depicted in varying green tones. Percentages of PROTACs tested in *in vivo* models are shown in the magenta-shaded slices: the percentages of degraders tested in different animal species and by means of alternative routes of administration (RoA) are presented (NHP: nonhuman primates; PO: oral RoA; IV: intravenous RoA; IP: intraperitoneal RoA; In CNS: both intracerebroventricular and stereotaxic RoAs; SC: subcutaneous RoA). Blue-shaded slices are dedicated to the various clusterings defined based on all cell lines used across available studies. Here, percentages refer to the amount of cell lines falling in each cluster relative to the total number of cell lines used in all available publications (∼60 lines). Cell line duplicates were retained and counted as separate instances (e.g., HEK293 cells could be found in multiple studies). (B) Details about cell lines clusters. Non-CNS cells represent the majority of instances, and Human Immortalized is the most crowded category of cells used for *in vitro* cell-based assays. (C) Focus on in vivo data. Here, percentages do not refer to the total 30 degraders but to all instances of animal models (left pie chart) and routes of administration (right chart) used across available publications. Mice and oral RoA were the most employed, respectively. (D) BBB permeability of the total 11 PROTACs tested *in vivo*. The 20% of these degraders (2/11) was not quantified in the brain of animal models after administration, while for the remaining 80% of degraders (i.e., 9/11, quantified in animal brain samples), 50% has PO administration, 20% IV and 10% (1 out of 9 degraders) is administered via SC route. This 9 PROTACs account for the 30% of degraders with demonstrated BBB penetration with respect to the collected 30 compounds from this work, as reported in panel A.

Concerning *in vitro* data for BBB passage across available publications, we found that only three degraders were tested with *in vitro* BBB models—two via a PAMPA-BBB (PROTAC 1, PT-65)^63,64^ and one with a Transwell-based coculture of mouse brain endothelial cells and human neuroblastoma cells (T3).^65^ In particular, T3—a dual degrader against α-synuclein and tau for the treatment of AD and PD—was added to the upper chamber of the Transwell-based coculture BBB model, and its fluorescence emission in SH-SY5Y was measured in the bottom compartment. Cell membrane permeability assays—a Caco-2 and a MDCK-MDR1 system—were used for other two degraders (XL01126, NX-5948).^61,66^ In summary, only five degraders were evaluated for cell permeability by using *in vitro* methods (Figure 5A). The remaining degraders were evaluated, *in vitro*, for BBB-unrelated aspects only, such as degradation efficiency, proteasome engagement, and cytotoxicity (data not shown). Overall, we counted–across all available studies—approximately 60 cell lines that were used for *in vitro* compound characterization (including the aforementioned membrane permeability tests) (Figure 5A). We grouped these cell lines—depending on their provenance—in CNS cells, non-CNS cells, and neuronal-like (non-CNS) cells, as well as into 7 clusters based on organism (human, mouse, rat, dog) and category (immortalized, primary, stem) (Figure 5A, B and ). Notably, most of the cell lines used (65%) originated from non-CNS tissues, which raises important concerns about their suitability for evaluating degraders designed to act within the brain. Such models may fail to capture critical neuronal or glial biology, potentially leading to misleading conclusions about target engagement, toxicity, or cellular responses—challenges that emerge even before addressing the additional barrier of BBB penetration.

Eleven PROTACs (37%) were tested on one or more *in vivo* models, most commonly mice (70% of cases), followed by rats (20%) and nonhuman primates (NHPs, 10%) (Figure 5C). Among all trials, the oral route (PO) was the most popular administration route (47% of cases), followed by the intravenous (IV, 24%), intraperitoneal (IP, 12%) and subcutaneous (SC, 6%) (Figure 5C). BBB permeability was confirmed in 9 out of these 11 PROTACs based on direct quantification in brain tissue from animal models (C004019, I3, KH1, T3, XL01126, NX-5948, CFT1946, ARV-102, and 20).^13,60,61,65–69^ We considered these degraders as CNS-penetrating compounds. Six of them are orally bioavailable (I3, XL01126, NX-5948, CFT1946, ARV-102, 20), two were administered via intravenous injection (KH1, T3) and the remaining one via subcutaneous injection (C004019) (Figure 5D). The remaining 2/11 compounds (C8 and PT-65) were not considered as brain-penetrant because they were not directly quantified in brain samples. As a matter of fact, C8 was administered intraperitoneally, but only behavioral tests were performed afterward. PT-65, on the other hand, was administered via stereotaxic injection, meaning that the BBB was bypassed. We noticed that, despite being tested with a PAMPA-BBB system, PROTAC 1 was not investigated *in vivo.* On the other hand, T3 was tested in an *in vitro*, cell-based BBB system as well as in a mouse model, and was ultimately detected in brain samples.

A notable finding is that, based on the unbound brain-to-plasma concentration ratio (wherever provided), CNS penetration fell greatly below the ranges typically considered optimal for small-molecule CNS drugs.^57^ However, this lower free concentration implies that PROTACs may not require high occupancy to be effective, leveraging their catalytic mechanism to operate at substoichiometric concentrations.^4^ Indeed, this is corroborated by pharmacodynamic data reported in the original studies: robust target degradation in brain tissue (assessed via Western blot or immunohistochemistry) was explicitly demonstrated for compounds C004019, KH1, T3, and 20, confirming that the measured exposure is sufficient to drive functional knockdown in the CNS. A further explanation on this apparent paradox can be found in the intrinsic chemical nature of PROTACs, which might lead to these molecules being sequestered by tissues and proteins due to high nonspecific binding.

Figure 6A reports the chemical structures of the nine BBB-penetrant PROTACs—E3 ligand in blue, protein of interest (POI) warhead in pink, and linker in gray. In the table that follows these structures, we also listed—apart from targeted E3 ligase and POI—the employed route of administration (RoA). Moreover, we calculated a series of widely used 2D molecular descriptors that can be easily and rapidly obtained.^70,71^ These calculated 2D descriptors included hydrophobicity-related metrics (MW; nC), polarity descriptors (nHDon, nHAcc, and TPSA), and measures of molecular flexibility (PHI, nRotB). To capture ionization behavior under physiological conditions, we also incorporated the strongest acidic and basic calculated p*K*_a_ values.

**Figure 6:**
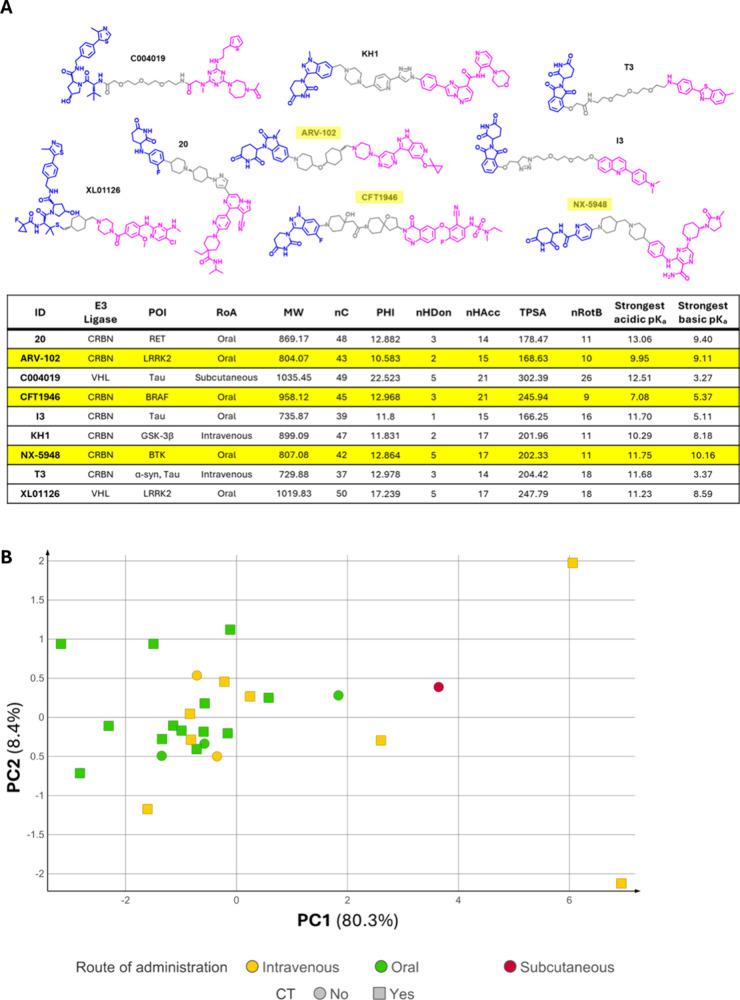
(A) BBB-permeable PROTACs (top figure). The E3 ligand is depicted in blue, linker in gray, and warhead in magenta. E3 ligand, target (POI), route of administration (RoA) and the main physicochemical properties of each degrader are reported in the bottom table. Degraders currently in clinical trials are highlighted in yellow. POI: protein of interest (target); RoA: route of administration; MW: molecular weight; nC: number of carbon atoms; PHI: Kier’s flexibility index; nHDon: number of hydrogen bond donors; nHAcc: number of hydrogen bond acceptors; TPSA: topological polar surface area; nRotB: number of rotatable bonds. (B) PCA plot of 2D physicochemical properties of PROTACs of interest: intravenously injected degraders are depicted in yellow (10 compounds), orally bioavailable ones are reported in green (16 molecules), and the remaining subcutaneous degrader is in dark red. PROTACs in clinical trials are square-shaped (CT, 21 compounds).

Figure 6A highlights that two BBB-penetrant PROTACs (C004019, XL01126) recruit the VHL E3 ligase, while the remaining ones use CRBN. This distinction is reflected by their molecular weights, with VHL-based PROTACs being generally larger. Predicted p*K*_a_ values reveal that none of the compounds but CFT1946 are expected to carry a net negative charge at physiological pH, whereas five are predicted to be at least partially positively charged (20, ARV-102, KH1, NX-5948, and XL01126). The number of hydrogen bond donors (nHDon) is typically below five, with only three compounds—C004019, NX-5948, and XL01126—reaching this threshold. In contrast, the number of hydrogen bond acceptors (nHAcc) is remarkably high, ranging from 14 to 21, which consequently results in elevated topological polar surface area (TPSA) values—always exceeding the CNS MPO threshold of 120 Å^2^.^57^ All analyzed PROTACs exhibit pronounced conformational flexibility, with the two VHL-based molecules showing the highest degree of flexibility within the series—as expressed by their PHI and nRotB values.

Finally, we compared the panel of the selected 2D physicochemical descriptors (MW, nC, PHI, nHDon, nHAcc, TPSA, and nRotB) for the nine BBB-permeable PROTACs with those we calculated for a set of PROTACs in clinical trials (noting that CFT1946 and NX-5948 were already included in this set) (). These degraders did not necessarily include CNS-active compounds, though they were addressed as examples of PROTACs with favorable ADME profile. Importantly, they are not the only degraders currently in clinical trials, but the ones for which a SMILES code was available at the time of data collection. Compounds were colored based on the RoA and shaped based on their development phase—namely, whether in clinical trial (CT) or not. As expected, the traditional 2D properties accounted for herein did not segregate the nine BBB-penetrant PROTACs into distinct groups, as shown by the principal component analysis (PCA) (Figure 6B and ). This outcome reinforces the notion that 2D descriptors alone are insufficient to capture the structural nuances of bRo5 molecules such as PROTACs, and that 3D descriptors are likely to provide a more informative framework for understanding their BBB permeability and overall behavior.^72^

Nevertheless, it should be noted that increasing evidence supports alternative entry mechanisms of bRo5 compounds other than passive diffusion. For instance, a recent study by Wang and colleagues identified the membrane receptor CD36—which is also expressed on BECs—as a key mediator that binds to various degraders (including ARV-110) and facilitates their endocytosis.^73^ Collectively, these findings open new perspectives for PROTAC development, suggesting that chemical features enhancing receptor-mediated uptake in the CNS should be integrated alongside traditional physicochemical optimization.

## Concluding Remarks

Despite growing interest in using PROTACs to treat neurodegenerative diseases, no established strategy currently exists for designing and evaluating molecules that can cross the BBB and act within the CNS. One way to begin formulating design guidelines for BBB-permeable PROTACs is to examine compounds for which BBB penetration has been unequivocally demonstrated. To this end, we manually assembled a data set of published PROTACs developed for CNS targets and analyzed the applied strategies. To enable a critical assessment of the methods used in these studies, we first provided an overview of BBB physiology along with the computational and experimental approaches currently employed to evaluate BBB accessibility.

Our analysis revealed that only a minority of the papers assessed PROTACs’ BBB permeability—typically through simplistic and outdated *in vitro* models. Less than one-half of the studies employed animal models, but rodent BBB properties significantly diverge from humans, thus limiting their predictive value. Moreover, the estimated *K*_*p,uu*_ and free brain concentrations were either not determined or found to be outside the typical range of small-molecule CNS drugs. It remains elusive whether the retrieved PROTACs could still be called CNS-active despite this evidence, even though many works cited herein highlighted a substantial target degradation efficiency in brain tissues. Overall, our analysis highlights an important gap: considerations of BBB permeability are still limited in the development of CNS-targeted PROTACs. This trend echoes earlier approaches adopted in medicinal chemistry, when the focus was placed primarily on optimizing activity, while ADME properties received less attention.

We also underscored another limitation in current PROTAC design for BBB-targeted applications: the widespread use of nonbrain cell lines to assess degradation. These models can yield misleading conclusions—primarily, the activity observed in cells unrelated to the CNS does not necessarily translate to efficacy within the brain parenchyma. Similarly, reliance on immortalized cell lines, which often lack sufficient human relevance to reflect the physiology of the intended target cells, represents an additional challenge. It is also important to recognize that compound-induced cytotoxicity in non-CNS cells may not mirror the responses observed in the neural cell types. However, because more solid models also need to be more standardized and scalable to reach a broader audience, less sophisticated—though high-throughput and practical—platforms keep being essential to foster CNS drug discovery.

In practice, improvements in any step of the current PROTAC design for BBB-targeted applications are required. Future *in silico* perspectives should encompass the use of protocols aimed at defining the molecular behavior of PROTACs in different simulated environments. These strategies hold promise for unmasking chameleonicity, that stands among the dynamic descriptors that can account for the permeability of PROTACs across the BBB. A key priority is the generation of robust, nonanimal data on BBB permeability that should enhance early stage screening and help reducing attrition in CNS drug development.

From the available literature, it emerges that human iPSCs represent a promising resource to generate BBB-relevant cells with efflux transporter profiles closer to the human *in vivo* scenario than animal-based models or noncerebral cell lines.^40^ Microfluidic devices—considering the increasing availability of more standardized, commercially available platforms—are good candidate models to (a) integrate iPSC-derived NVU components and (b) to replace current high-throughput systems for BBB permeability assessment. The data collected in these improved models would also enable the construction of more representative training sets for developing accurate *in silico* predictions of the CNS activity of PROTACs. Regarding *in vivo* studies, any claim of CNS activity should clearly specify whether it is supported by pharmacokinetic data, pharmacodynamic evidence (e.g., behavioral or imaging outcomes), or both.

Overall, future efforts to advance early drug discovery of PROTACs for NDDs should prioritize the development of more robust, reproducible, and human-relevant BBB models. Improving the reliability of these systems is essential for generating high-quality data. Although AI technologies hold great promise for identifying new principles governing BBB penetration, their success largely depends on access to large, well-curated experimental data sets—resources that are currently limited or of insufficient quality. Strengthening the experimental foundation will, therefore, be a critical step toward enabling meaningful AI-driven insights in this area.

## Supplementary Material







## Notes

Figures, tables, methods, and additional references (PDF)
Table S1 (XLSX)
Table S5 (XLSX)

## Abbreviations

NDD: neurodegenerative disorder
AD: Alzheimer’s disease
PD: Parkinson disease
FTD: frontotemporal dementia
HD: Huntington’s disease
ALS: amyotrophic lateral sclerosis
Ro5: Rule-of-Five
PPI: protein–protein interaction
BBB: blood–brain barrier
PROTAC: proteolysis targeted chimera
UPS: ubiquitin-proteasome system
POI: protein of interest
TC: ternary complex
MoA: mode of action
TPD: targeted protein degradation
bRo5: beyond Rule-of-Five
CNS: central nervous system
BEC: brain endothelial cell
NVU: neurovascular unit
IB: intestinal barrier
JAM: junctional adhesion molecule
TEER: transendothelial electrical resistance
ZO: zonula occludens
CMT: carrier-mediated transport
GLUT: glucose transporter
LAT: L-amino acid transporter
CAT: cationic amino acid transporter
RMT: receptor-mediated transport
P-gp: P-glycoprotein
BCRP: breast cancer resistance protein
MRP: multidrug resistance-associated protein
MFSD2A: major facilitator superfamily domain containing 2A
DHA: docosahexaenoic acid
CX: connexin
nHDon/HBD: number of hydrogen bond donors
TPSA: topological polar surface area
QSPR: quantitative structure–property relationships
MW: molecular weight
IMHB: intramolecular hydrogen bond
MD: molecular dynamics
PAMPA: parallel artificial membrane permeability assay
HTS: high-throughput screening
iPSC: induced pluripotent stem cells
3D-DIV: three-dimensional dynamic in vitro system
LC-MS: liquid-chromatography–mass spectrometry
NHP: nonhuman primate
PO: per os (oral administration route)
IV: intravenous
IP: intraperitoneal
SC: subcutaneous
RoA: route of administration
nC: number of carbon atoms
nHAcc/HBA: number of hydrogen acceptors
PHI: Kier’s flexibility index
nRotB: number of rotatable bonds
VHL: Von Hippel-Lindau
CRBN: Cereblon
PCA: principal component analysis

## References

[1] Witters, H. ; Verstraelen, S. ; Aerts, L. ; Miccoli, B. ; Delahanty, A. ; Dura, A. ; Gribaldo, L. ; Whelan, M. Advanced Non-Animal Models in Biomedical Research; Publications Office of the European Union, 2021; JRC124723.

[2] Lipinski C.
A., Lombardo F., Dominy B. W., Feeney P. J. (1997). Experimental and
Computational Approaches to Estimate Solubility and Permeability in
Drug Discovery and Development Settings. Adv.
Drug Delivery Rev..

[3] Ding Y., Fei Y., Lu B. (2020). Emerging New
Concepts of Degrader Technologies. Trends Pharmacol.
Sci..

[4] Békés M., Langley D. R., Crews C. M. (2022). PROTAC Targeted
Protein Degraders:
The Past Is Prologue. Nat. Rev. Drug Discovery.

[5] Bhatia S., Singh M., Singh T., Singh V. (2023). Scrutinizing the Therapeutic
Potential of PROTACs in the Management of Alzheimer’s Disease. Neurochem. Res..

[6] Walz C., Spiske M., Walter M., Keller B. L., Mezler M., Hoft C., Pohlki F., Vukelić S., Hausch F. (2025). Macrocyclization as a Strategy for
Kinetic Solubility
Improvement: A Comparative Analysis of Matched Molecular Pairs. J. Med. Chem..

[7] Brudy C., Ruijsenaars E., Meyners C., Sugiarto W. O., Achaq H., Spiske M., Buffa V., Springer M., Repity M., Weller A., Haferkamp U., Pless O., Muschong P., Miltner D., Mezler M., Schmidt M. V., Riniker S., Hausch F. (2025). Linker Modification
Enables Control of Key Functional
Group Orientation in Macrocycles. J. Med. Chem..

[8] Price E., Weinheimer M., Rivkin A., Jenkins G., Nijsen M., Cox P. B., DeGoey D. (2024). Beyond Rule of Five and PROTACs in
Modern Drug Discovery: Polarity Reducers, Chameleonicity, and the
Evolving Physicochemical Landscape. J. Med.
Chem..

[9] Hasegawa M., Arai T., Nonaka T., Kametani F., Yoshida M., Hashizume Y., Beach T. G., Buratti E., Baralle F., Morita M., Nakano I., Oda T., Tsuchiya K., Akiyama H. (2008). Phosphorylated TDP-43 in Frontotemporal
Lobar Degeneration
and Amyotrophic Lateral Sclerosis. Ann. Neurol..

[10] Wang J. Z., Wu Q., Smith A., Grundke-Iqbal I., Iqbal K. (1998). τ Is Phosphorylated
by GSK-3 at Several Sites Found in Alzheimer Disease and Its Biological
Activity Markedly Inhibited Only after It Is Prephosphorylated by
A-Kinase. FEBS Lett..

[11] Zündorf G., Reiser G. (2011). Calcium Dysregulation
and Homeostasis of Neural Calcium
in the Molecular Mechanisms of Neurodegenerative Diseases Provide
Multiple Targets for Neuroprotection. Antioxid.
Redox Signal..

[12] Kinscherf N. A., Pehar M. (2022). Role and Therapeutic Potential of RAGE Signaling in Neurodegeneration. Curr. Drug Targets.

[13] Holmqvist A., Kocaturk N. M., Duncan C., Riley J., Baginski S., Marsh G., Cresser-Brown J., Maple H., Juvonen K., Sathe G., Morrice N., Sutherland C., Read K. D., Farnaby W. (2025). Discovery of a CNS
Active GSK3 Degrader
Using Orthogonally Reactive Linker Screening. Nature Communications 2025 16:1.

[14] Pandit R., Chen L., Götz J. (2020). The Blood-Brain
Barrier: Physiology
and Strategies for Drug Delivery. Adv. Drug
Delivery Rev..

[15] McConnell H. L., Mishra A. (2022). Cells of the Blood-Brain Barrier:
An Overview of the
Neurovascular Unit in Health and Disease. Methods
Mol. Biol..

[16] Abou
Diwan M., Lahimer M., Bach V., Gosselet F., Khorsi-Cauet H., Candela P. (2023). Impact of Pesticide Residues on the
Gut-Microbiota-Blood-Brain Barrier Axis: A Narrative Review. Int. J. Mol. Sci..

[17] Pfau S. J., Langen U. H., Fisher T. M., Prakash I., Nagpurwala F., Lozoya R. A., Lee W. C. A., Wu Z., Gu C. (2024). Characteristics
of Blood-Brain Barrier Heterogeneity between Brain Regions Revealed
by Profiling Vascular and Perivascular Cells. Nat. Neurosci..

[18] Kadry, H. ; Noorani, B. ; Cucullo, L. A Blood-Brain Barrier Overview on Structure, Function, Impairment, and Biomarkers of Integrity. Fluids Barriers CNS 2020, 17 (1). 10.1186/s12987-020-00230-3. PMC7672931 33208141

[19] Sabbagh, M. F. ; Heng, J. S. ; Luo, C. ; Castanon, R. G. ; Nery, J. R. ; Rattner, A. ; Goff, L. A. ; Ecker, J. R. ; Nathans, J. Transcriptional and Epigenomic Landscapes of CNS and Non-CNS Vascular Endothelial Cells. Elife 2018, 7. 10.7554/eLife.36187. PMC6126923 30188322

[20] Andreone B. J., Chow B. W., Tata A., Lacoste B., Ben-Zvi A., Bullock K., Deik A. A., Ginty D. D., Clish C. B., Gu C. (2017). Blood-Brain Barrier
Permeability Is Regulated by Lipid Transport-Dependent
Suppression of Caveolae-Mediated Transcytosis. Neuron.

[21] Hall C. N., Reynell C., Gesslein B., Hamilton N. B., Mishra A., Sutherland B. A., O'Farrell F. M., Buchan A. M., Lauritzen M., Attwell D. (2014). Capillary Pericytes Regulate Cerebral Blood Flow in
Health and Disease. Nature.

[22] Sofroniew M. V., Vinters H. V. (2010). Astrocytes: Biology
and Pathology. Acta Neuropathol..

[23] Thurgur H., Pinteaux E. (2019). Microglia in the Neurovascular
Unit: Blood-Brain Barrier-Microglia
Interactions After Central Nervous System Disorders. Neuroscience.

[24] Bagchi S., Chhibber T., Lahooti B., Verma A., Borse V., Jayant R. D. (2019). In-Vitro Blood-Brain
Barrier Models for Drug Screening
and Permeation Studies: An Overview. Drug Des.
Devel. Ther..

[25] Yuan, Y. ; Sun, J. ; Dong, Q. ; Cui, M. Blood-Brain Barrier Endothelial Cells in Neurodegenerative Diseases: Signals from the “Barrier.” Front. Neurosci. 2023, 17. 10.3389/fnins.2023.1047778. PMC9998532 36908787

[26] Knox E. G., Aburto M. R., Clarke G., Cryan J. F., O’Driscoll C.
M. (2022). The Blood-Brain
Barrier in Aging and Neurodegeneration. Mol.
Psychiatry.

[27] Pulgar, V. M. Transcytosis to Cross the Blood Brain Barrier, New Advancements and Challenges. Front. Neurosci. 2019, 12. 10.3389/fnins.2018.01019. PMC6337067 30686985

[28] Hang Z., Zhou L., Xing C., Wen Y., Du H. (2023). The Blood-Brain
Barrier, a Key Bridge to Treat Neurodegenerative Diseases. Ageing Res. Rev..

[29] Kang D. E., Pietrzik C. U., Baum L., Chevallier N., Merriam D. E., Kounnas M. Z., Wagner S. L., Troncoso J. C., Kawas C. H., Katzman R., Koo E. H. (2000). Modulation of Amyloid
β-Protein Clearance and Alzheimer’s Disease Susceptibility
by the LDL Receptor-Related Protein Pathway. J. Clin. Invest..

[30] Mooradian A. D. (1988). Effect
of Aging on the Blood-Brain Barrier. Neurobiol.
Aging.

[31] Xiong B., Wang Y., Chen Y., Xing S., Liao Q., Chen Y., Li Q., Li W., Sun H. (2021). Strategies
for Structural Modification of Small Molecules to Improve Blood-Brain
Barrier Penetration: A Recent Perspective. J.
Med. Chem..

[32] Xu Z., Ji Y., Wen C., Gan J., Chen Z., Li R., Lin X., Dou J., Wang Y., Liu S., Shi Z., Wu H., Lu H., Chen H. (2025). Tracer Kinetic Model
Detecting Heterogeneous
Blood-Brain Barrier Permeability to Water and Contrast Agent in Alzheimer’s
Disease and Dementia with Lewy Bodies. Alzheimer’s
& Dementia.

[33] Wager T. T., Hou X., Verhoest P. R., Villalobos A. (2016). Central Nervous System Multiparameter
Optimization Desirability: Application in Drug Discovery. ACS Chem. Neurosci..

[34] Tsuji A., Tamai I., Sakata A., Tenda Y., Terasaki T. (1993). Restricted
Transport of Cyclosporin A across the Blood-Brain Barrier by a Multidrug
Transporter, P-Glycoprotein. Biochem. Pharmacol..

[35] Caron G., Kihlberg J., Ermondi G. (2019). Intramolecular Hydrogen Bonding:
An Opportunity for Improved Design in Medicinal Chemistry. Med. Res. Rev..

[36] Poongavanam V., Wieske L. H. E., Peintner S., Erdélyi M., Kihlberg J. (2024). Molecular Chameleons in Drug Discovery. Nat. Rev. Chem..

[37] Soliman Y., Al-khodor J., Yildirim Köken G., Mustafaoglu N. (2025). A Guide for
Blood-Brain Barrier Models. FEBS Lett..

[38] Kansy M., Senner F., Gubernator K. (1998). Physicochemical High Throughput Screening:
Parallel Artificial Membrane Permeation Assay in the Description of
Passive Absorption Processes. J. Med. Chem..

[39] Di L., Kerns E. H., Fan K., McConnell O. J., Carter G. T. (2003). High Throughput Artificial Membrane
Permeability Assay
for Blood-Brain Barrier. Eur. J. Med. Chem..

[40] Appelt-Menzel, A. ; Oerter, S. ; Mathew, S. ; Haferkamp, U. ; Hartmann, C. ; Jung, M. ; Neuhaus, W. ; Pless, O. Human IPSC-Derived Blood-Brain Barrier Models: Valuable Tools for Preclinical Drug Discovery and Development? Curr. Protoc. Stem Cell Biol. 2020, 55 (1). 10.1002/cpsc.122. 32956578

[41] Prieto P., Blaauboer B. J., De Boer A. G., Boveri M., Cecchelli R., Clemedson C., Coecke S., Forsby A., Galla H. J., Garberg P., Greenwood J., Price A., Tähti H. (2004). Blood-Brain
Barrier In Vitro Models and Their Application in Toxicology: The Report
and Recommendations of ECVAM Workshop 49. Alternatives
to Laboratory Animals.

[42] Cecchelli R., Berezowski V., Lundquist S., Culot M., Renftel M., Dehouck M. P., Fenart L. (2007). Modelling of the Blood - Brain Barrier
in Drug Discovery and Development. Nat. Rev.
Drug Discovery.

[43] Jin X., Luong T. L., Reese N., Gaona H., Collazo-Velez V., Vuong C., Potter B., Sousa J. C., Olmeda R., Li Q., Xie L., Zhang J., Zhang P., Reichard G., Melendez V., Marcsisin S. R., Pybus B. S. (2014). Comparison of MDCK-MDR1
and Caco-2 Cell Based Permeability Assays for Anti-Malarial Drug Screening
and Drug Investigations. J. Pharmacol. Toxicol.
Methods.

[44] Hayashi K., Nakao S., Nakaoke R., Nakagawa S., Kitagawa N., Niwa M. (2004). Effects of Hypoxia on Endothelial/Pericytic
Co-Culture Model of the
Blood-Brain Barrier. Regul. Pept..

[45] Chaulagain B., Gothwal A., Lamptey R. N. L., Trivedi R., Mahanta A. K., Layek B., Singh J. (2023). Experimental Models of In Vitro Blood-Brain
Barrier for CNS Drug Delivery: An Evolutionary Perspective. Int. J. Mol. Sci..

[46] Wasielewska J. M., Da Silva Chaves J. C., White A. R., Oikari L. E. (2020). Modeling the Blood-Brain
Barrier to Understand Drug Delivery in Alzheimer’s Disease. Alzheimer’s Disease: Drug Discovery.

[47] Hajal C., Offeddu G. S., Shin Y., Zhang S., Morozova O., Hickman D., Knutson C. G., Kamm R. D. (2022). Engineered Human
Blood-Brain Barrier Microfluidic Model for Vascular Permeability Analyses. Nat. Protoc..

[48] Wang Y. I., Abaci H. E., Shuler M. L. (2017). Microfluidic Blood-Brain
Barrier
Model Provides in Vivo-like Barrier Properties for Drug Permeability
Screening. Biotechnol. Bioeng..

[49] Park, T. E. ; Mustafaoglu, N. ; Herland, A. ; Hasselkus, R. ; Mannix, R. ; FitzGerald, E. A. ; Prantil-Baun, R. ; Watters, A. ; Henry, O. ; Benz, M. ; Sanchez, H. ; McCrea, H. J. ; Goumnerova, L. C. ; Song, H. W. ; Palecek, S. P. ; Shusta, E. ; Ingber, D. E. Hypoxia-Enhanced Blood-Brain Barrier Chip Recapitulates Human Barrier Function and Shuttling of Drugs and Antibodies. Nat. Commun. 2019, 10 (1). 10.1038/s41467-019-10588-0. PMC6565686 31197168

[50] Helms H. C., Abbott N. J., Burek M., Cecchelli R., Couraud P. O., Deli M. A., Förster C., Galla H. J., Romero I. A., Shusta E. V., Stebbins M. J., Vandenhaute E., Weksler B., Brodin B. (2016). In Vitro Models of
the Blood-Brain Barrier: An Overview of Commonly Used Brain Endothelial
Cell Culture Models and Guidelines for Their Use. Journal of Cerebral Blood Flow & Metabolism.

[51] Noorani B., Bhalerao A., Raut S., Nozohouri E., Bickel U., Cucullo L. (2021). A Quasi-Physiological
Microfluidic
Blood-Brain Barrier Model for Brain Permeability Studies. Pharmaceutics.

[52] Sharma S., Zhang Y., Akter K. A., Nozohouri S., Archie S. R., Patel D., Villalba H., Abbruscato T. (2023). Permeability
of Metformin across an In Vitro Blood-Brain Barrier Model during Normoxia
and Oxygen-Glucose Deprivation Conditions: Role of Organic Cation
Transporters (Octs). Pharmaceutics.

[53] Cui Y., Desevaux C., Truebenbach I., Sieger P., Klinder K., Long A., Sauer A. (2021). A Bidirectional
Permeability Assay
for beyond Rule of 5 Compounds. Pharmaceutics.

[54] Langley G. R. (2014). Considering
a New Paradigm for Alzheimer’s Disease Research. Drug Discovery Today.

[55] Konnova E. A., Swanberg M. (2018). Animal Models of Parkinson’s
Disease. Parkinson’s Disease: Pathogenesis
and Clinical Aspects.

[56] Hoshi Y., Uchida Y., Tachikawa M., Inoue T., Ohtsuki S., Terasaki T. (2013). Quantitative Atlas
of Blood-Brain Barrier Transporters,
Receptors, and Tight Junction Proteins in Rats and Common Marmoset. J. Pharm. Sci..

[57] Di L., Rong H., Feng B. (2013). Demystifying
Brain Penetration in
Central Nervous System Drug Discovery. J. Med.
Chem..

[58] NIH to prioritize human-based research technologies | National Institutes of Health (NIH). https://www.nih.gov/news-events/news-releases/nih-prioritize-human-based-research-technologies (accessed 2025–07–04).

[59] Wasielewska J. M., Da Silva Chaves J. C., White A. R., Oikari L. E. (2020). Modeling the Blood-Brain
Barrier to Understand Drug Delivery in Alzheimer’s Disease. Alzheimer’s Disease: Drug Discovery.

[60] Kreger B. T., Liang Y., Reilly N. M., Spears M. E., Scott W. A., Simard J. R., Li P., Chaturvedi P., Agafonov R. V., Stephenson J., Baddour J., Hart J. A., Bahadduri P. M., Riegel L., Cole K., Lobbardi R., Follmer N. E., Hurh E., Good A., Fitzgerald M. E., Patel J., Jackson K. L., Poling L. L., Phillips A. J., Nasveschuk C. G., Fisher S. L., Pollock R. M., Sowa M. E. (2026). CFT1946
Is an Orally Available Brain-Penetrant BRAF V600-Mutant Degrader That
Overcomes BRAF Inhibitor Resistance. Running Title CFT1946 Is an Oral
BRAF V600-Mutant Selective Degrader. Cancer
Res..

[61] Robbins D. W., Noviski M., Rountree R., Tan M., Brathaban N., Ingallinera T., Karr D. E., Kelly A., Konst Z., Ma J., Tenn-McClellan A., McKinnell J., Perez L., Guiducci C., Hansen G., Sands A. (2021). Nx-5948, a
Selective Degrader of BTK with Activity in Preclinical Models of Hematologic
and Brain Malignancies. Blood.

[62] ARV-102|CAS|DC Chemicals. https://www.dcchemicals.com/product_show-arv-102.html (accessed 2025-12-11).

[63] Guardigni M., Pruccoli L., Santini A., Simone A. De, Bersani M., Spyrakis F., Frabetti F., Uliassi E., Andrisano V., Pagliarani B., Fernández-Gómez P., Palomo V., Bolognesi M. L., Tarozzi A., Milelli A. (2023). PROTAC-Induced
Glycogen Synthase Kinase 3β Degradation as a Potential Therapeutic
Strategy for Alzheimer’s Disease. ACS
Chem. Neurosci..

[64] Qu L., Li S., Ji L., Luo S., Ding M., Yin F., Wang C., Luo H., Lu D., Liu X., Peng W., Kong L., Wang X. (2021). Discovery
of PT-65
as a Highly Potent and Selective Proteolysis-Targeting Chimera Degrader
of GSK3 for Treating Alzheimer’s Disease. Eur. J. Med. Chem..

[65] Zhu W., Zhang W., Chen J., Tong Y., Xu F., Pang J. (2024). Discovery of Effective
Dual PROTAC Degraders for Neurodegenerative
Disease-Associated Aggregates. J. Med. Chem..

[66] Liu X., Kalogeropulou A. F., Domingos S., Makukhin N., Nirujogi R. S., Singh F., Shpiro N., Saalfrank A., Sammler E., Ganley I. G., Moreira R., Alessi D. R., Ciulli A. (2022). Discovery of XL01126:
A Potent, Fast, Cooperative,
Selective, Orally Bioavailable, and Blood-Brain Barrier Penetrant
PROTAC Degrader of Leucine-Rich Repeat Kinase 2. J. Am. Chem. Soc..

[67] Wang W., Zhou Q., Jiang T., Li S., Ye J., Zheng J., Wang X., Liu Y., Deng M., Ke D., Wang Q., Wang Y., Wang J. Z. (2021). A Novel Small-Molecule
PROTAC Selectively Promotes Tau Clearance to Improve Cognitive Functions
in Alzheimer-like Models. Theranostics.

[68] Liang M., Gu L., Zhang H., Min J., Wang Z., Ma Z., Zhang C., Zeng S., Pan Y., Yan D., Shen Z., Huang W. (2022). Design, Synthesis,
and Bioactivity
of Novel Bifunctional Small Molecules for Alzheimer’s Disease. ACS Omega.

[69] Orsi D. L., Lazarski K. E., Improgo R., Agafonov R. V., Ahn J. Y., Baddour J., Cassidy K., Chaturvedi P., Cole K. S., Deibler R. W., Elam W. A., Fitzgerald M. E., Garza V. J., Good A., Hulton C. H., Isasa M., Jackson K. L., Li P., Liang Y., Michael R. E., O’Shea M. W., Moustakim M., Perino S., Rahman F., Schnaderbeck M. J., Stone N. P., Tillotson B., Veits G. K., Vogelaar A., Yap J. L., Yu R. T., Huang H., Henderson J. A. (2025). Discovery
of an Orally Bioavailable,
CNS Active Pan-Mutant RET Kinase Heterobifunctional Degrader. RSC Med. Chem..

[70] Ermondi G., Garcia-Jimenez D., Caron G. (2021). PROTACs and Building Blocks: The
2D Chemical Space in Very Early Drug Discovery. Molecules.

[71] Apprato G., Caron G., Deshmukh G., Garcia-Jimenez D., Haid R. T. U., Pike A., Reichel A., Rynn C., Zhang D., Wittwer M. B. (2025). Finding a Needle in the Haystack:
ADME and Pharmacokinetics/Pharmacodynamics Characterization and Optimization
toward Orally Available Bifunctional Protein Degraders. Expert Opin. Drug Discovery.

[72] Apprato G., Poongavanam V., Garcia Jimenez D., Atilaw Y., Erdelyi M., Ermondi G., Caron G., Kihlberg J. (2024). Exploring the Chemical
Space of Orally Bioavailable PROTACs. Drug Discovery
Today.

[73] Wang Z., Pan B. S., Manne R. K., Chen J., Lv D., Wang M., Tran P., Weldemichael T., Yan W., Zhou H., Martinez G. M., Shao J., Hsu C. C., Hromas R., Zhou D., Qin Z., Lin H. K., Li H. Y. (2025). CD36-Mediated Endocytosis of Proteolysis-Targeting Chimeras. Cell.

