# Supplementary material for: BBB-Permeable PROTACs: Where Do We Stand?
Source: ACS Med Chem Lett. 2026 Mar 5;17(4):776–88. doi: 10.1021/acsmedchemlett.5c00768 (PMC13071617; doi:10.1021/acsmedchemlett.5c00768)
Supplement: Supplementary file 1 [file ml5c00768_si_001.pdf]

BBB-Permeable PROTACs: Where Do We Stand?

Serena Francisco, Giulia Apprato, Matteo Rossi Sebastiano, Giuseppe Ermondi, Giulia Caron*

Affiliations

Department of Molecular Biotechnology and Health Sciences, MedChemBeyond Lab, University of Torino, Torino, Italy.

* Corresponding Author, giulia.caron@unito.it

Supplementary information

1. Figures
2. Tables
3. Methods
4. Additional references

1 Figures

<u>**Figure S1**</u>. ***Mode of action of PROteolysis TArgeting Chimeras (PROTACs).*** *Since their development, PROTACs targeting about 130 different proteins have been designed and a few of them managed to reach clinical trials.[1] PROTACs can exploit three major classes of E3 ligases, namely the cellular inhibitor of apoptosis protein (cIAP), the Von Hippel–Lindau (VHL) and cereblon (CRBN).[2] Unfortunately, this is just a small set, thus the amount of targets that can be sent to UPS-dependent degradation is still limited.[3] Moreover, the identification of tissue-specific E3 ligases emerges as a pivotal task in order to reduce degradation in off-target compartments and unwanted toxic effects.*

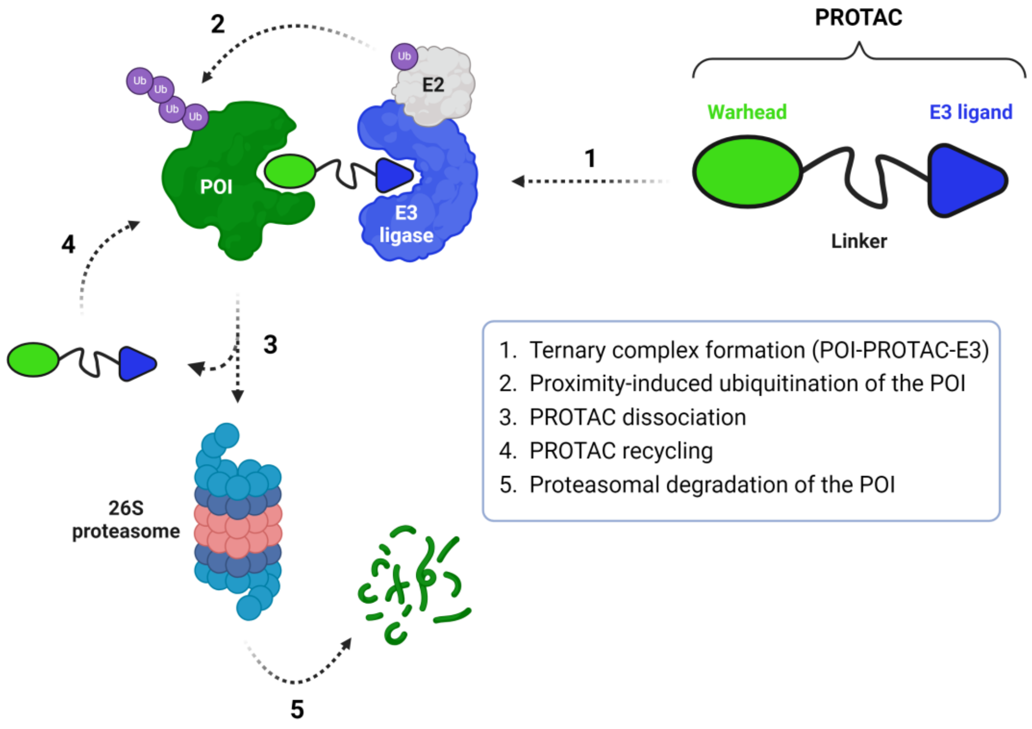

<u>Figure S2</u>. *Principal component analysis (PCA) loadings.* *Loadings from the PCA reported in Figure 6, which was performed using DataWarrior, are presented below.*

Variable	PC1	PC2
MW	0.400554	-0.30448
nC	0.363554	-0.49601
nHAcc	0.372174	0.089014
nHDon	0.3243	0.721188
TPSA(Tot)	0.399606	0.322176
nRotB	0.372608	-0.09505
PHI	0.406423	-0.14283

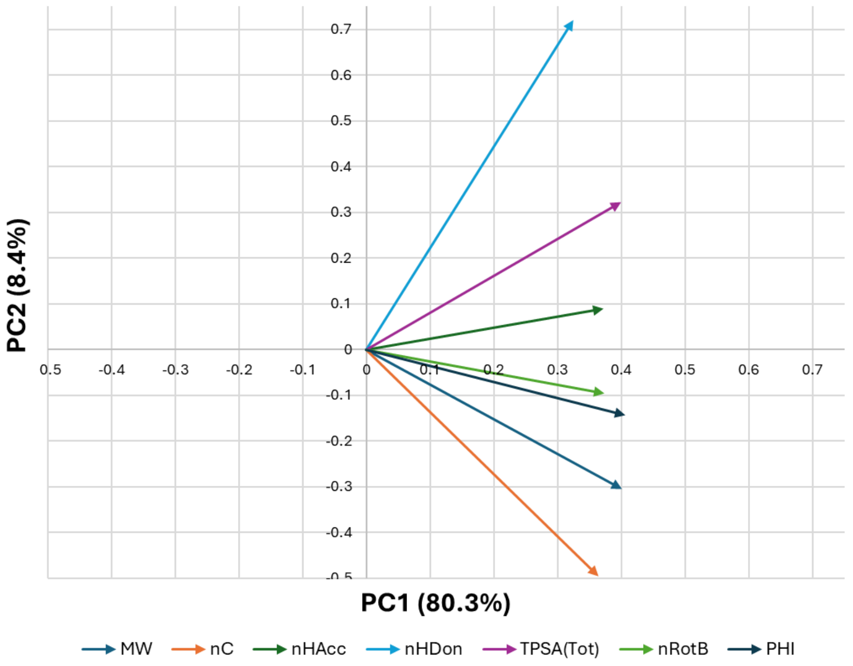

2 Tables

Table S1 (separate Excel file). *A set of proteins found at the membrane of brain endothelial cells (BECs) is reported. For each protein, we annotated: (1) protein category, (2) human gene name, (3) human protein name, (4) human protein length, (5) known endogenous substrates (if applicable) (6) UniProt ID, (7) number of UniProt-related PDB entries, (8) ligands from DrugBank, (9) ligands from ChEMBL, (10) relevant literature references and (11) number of reported BBB-related studies. Since this list was curated from multiple species, it should be interpreted with caution. We believe this resource could represent a valuable piece of information for drug discovery, as it highlights the state-of-art about research on BBB components, pointing out untapped targets too.*

Table S2. ***Overview and comparison of cell types available for BBB modelling.*** *Examples of immortalized BECs lines include hCMEC/D3 and bEnd3 (a list of human and non-human BECs is reported in many available publications).[4] Human iPSCs-derived BECs (iBECs) can be – for example – AD patient-derived and display better BBB markers. Human iPSCs from the same patient can be derived into BECs as well as into other NVU components, thus enabling to have an isogenic multi-cell BBB model.[5,6] However, they are highly variable and de-differentiation typically occurs rapidly (short-term studies).[7] A relevant drawback characteristic of all three cell types is the incapacity of modelling NDDs with spontaneous onset.*

	Primary cells	***Stem cells***	***Immortalized cells***
Source	Biopsy/tissue collection	• Biopsy/tissue collection<br>• Cell reprogramming	• Transformation/immortalization of primary cells<br>• Tumour biopsy
Similarity to in vivo counterparts	High	High (upon differentiation)	Low to moderate (may lose tissue-specific features)
Required expertise (e.g., isolation, risk of contamination)	High	High	Low to moderate
Maintenance costs	High	High	Low
Lifespan	Short	Long (though progressive loss of original phenotype possible)	Unlimited (but may genetically drift over time due to instability)
Disease modelling	Yes (though for short-term studies)	Yes (especially genetic diseases, upon differentiation)	Limited (due to altered phenotype)
Specific drawbacks in BBB modelling (e.g., protein expression, barrier integrity, TEER values)	• Require post-mortem samples (limited availability)<br>• Animal-derived cells differ from human cells<br>• Donor-related variability	• Can require complex differentiation protocols<br>• Genetic instability in long-term culture	• Animal-derived cells differ from human cells<br>• Transformation processes can alter the original phenotype
Scalability for drug discovery	Low	Medium	High

<u>Table S3</u>. *Selected PROTAC degraders with their target and SMILES codes and reference (see Additional References in this file).* *For studies presenting more than one compound, the one with the best PD and PK profile (according to the authors) was selected. Where no clear discrimination was made available, all degraders were retained. Names were assigned according to the original publication. For 6 compounds, the relative ID from the PROTAC-DB was assigned instead (PRODB_<serialnumber>), as no other name was made available.*

PROTAC name	Target	SMILES	Quantified in the CNS	Reference
**1**	Htt	Cc4ccc3nc(c2ccc(NCCOCCOCCNC(=O)[C@@H](CC(C)C)NC(=O)[C@H](O)[C@@H](N)Cc1ccccc1)cc2)sc3c4	No	8
**2**	Htt	CC(C)C[C@@H](NC(=O)[C@H](O)[C@@H](N)Cc1ccccc1)C(=O)NCCOCCOCCOc4ccc3nc(/N=N/c2ccc(N(C)C)cc2)sc3c4	No	8
**5**	α-syn	CC(C)C[C@H](NC(=O)[C@@H](O)[C@H](N)Cc1ccccc1)C(=O)NCCOCCOCCOCCNc4ccc(c3cc(c2cccc(Br)c2)n[nH]3)cc4	No	9
**20b**	KDM5	CC(C)c7c(c5ccc(CCN(C)CCCCCCc4cccc(C(=O)NCCOCCOCCOc2cccc3c(=O)n(C1CCC(=O)NC1=O)c(=O)c23)c4)cc5)[nH]c6c(C#N)cnn6c7=O	No	10
**23b**	KDM5	CC(C)c7c(c5ccc(CCN(C)CCCCCCc4cccc(C(=O)NCCCCCCOc2cccc3c(=O)n(C1CCC(=O)NC1=O)c(=O)c23)c4)cc5)[nH]c6c(C#N)cnn6c7=O	No	10
**2b**	α-syn	O=C(CNC1=CC=CC(C2=CC(C3=CC=C4OCOC4=C3)=N[NH]2)=C1)NCCOCCOCCOCCNC1=CC=CC2=C1C(=O)N(C1CCC(=O)NC1=O)C2=O	No	11
**8a**	α-syn	O=C8CCC(N7Cc6c(NC(=O)CCCc5cn(c4cccc(c3cc(c2ccc1OCOc1c2)[nH]n3)c4)nn5)cccc6C7=O)C(=O)N8	No	12
**C004019**	Tau	O=C(N1[C@H](C[C@@H](C1)O)C(NCC2=CC=C(C3=C(N=CS3)C)C=C2)=O)[C@H](C(C)(C)C)NC(COCCOCCOCCNC(CN(C4=NC(N5CCN(CCC5)C(C)=O)=NC(NCCC6=CC=CS6)=N4)C)=O)=O	Yes	13
**C8**	Tau	CN(C)C1=CC=C(S1)\C=C\C1=NC2=CC=C(C=C2N=C1)C(=O)NCCCCCCCCNC1=CC=CC2=C1C(=O)N(C1CCC(=O)N(C)C1=O)C2=O	No	14
**FMF-06-049**	Tau	O=C(CCC1=CC=C2C(=C1)[NH]C1=CC=NC=C12)NCCCCCCOC1=CC=CC2=C1C(=O)N(C1CCC(=O)NC1=O)C2=O	No	15
**I3**	Tau	CN(C)C1=CC=C(C=C1)C1=NC2=C(C=C1)C=C(OCCOCCOCCN1CC(COC3=C4C(=O)N(C5CCC(=O)NC5=O)C(=O)C4=CC=C3)N=N1)C=C2	Yes	16
**JH-XII-03-02**	LRRK2	CC1(OC2=CC=C3[NH]N=C(C4=CC(N5CCN(C(=O)CCOCCOCCOCCOCCNC6=CC=CC7=C6C(=O)N(C6CCC(=O)NC6=O)C7=O)CC5)=NC=N4)C3=C2)CC1	No	17
**JMF4560**	TDP-43	CN(C)C1=CC=C(C2=NC3=CC=C(OCCOCCOCCNC4=CC=CC5=C4C(=O)N(C4CCC(=O)NC4=O)C5=O)C=C3S2)C=C1	No	18
**KH1**	GSK-3β	CN1N=C(N2CCC(=O)NC2=O)C2=C1C=C(CN1CCN(CC3=CN=C(C=C3)C3=CN(N=N3)C3=CC=C(C=C3)C3=CN4N=CC=C(C(=O)NC5=C(C=CN=C5)N5CCOCC5)C4=N3)CC1)C=C2	Yes	19
**PG21**	GSK-3β	CNC(=O)C1=C(OCCCCCCCCN2C=C(COC3=CC=CC4=C3C(=O)N(C3CCC(=O)NC3=O)C4=O)N=N2)N=C(C2=CC=NC(NC(=O)C3CC3)=C2)S1	No	20
**PRODB_1570**	α-syn	CNC1=CC=C(C2=NC3=CC=C(OCCOCCOCC(=O)N[C@H](C(=O)N4C[C@H](O)C[C@H]4C(=O)N[C@@H](C)C4=CC=C(C5=C(C)N=CS5)C=C4)C(C)(C)C)C=C3S2)C=C1	No	21
**PRODB_1571**	α-syn	CNC1=CC=C(C2=NC3=CC=C(OCCOCCOCCOCCOCCOCC(=O)N[C@H](C(=O)N4C[C@H](O)C[C@H]4C(=O)N[C@@H](C)C4=CC=C(C5=C(C)N=CS5)C=C4)C(C)(C)C)C=C3S2)C=C1	No	21

**PRODB_1572**	α-syn	CNC1=CC=C(C2=NC3=CC=C(OCCOCCOCCOCCOCCOCCOCC(=O)N[C@H](C(=O)N4C[C@H](O)C[C@H]4C(=O)N[C@@H](C)C4=CC=C(C5=C(C)N=CS5)C=C4)C(C)(C)C)C=C3S2)C=C1	No	21
**PRODB_1573**	α-syn	CC1=C(C2=CC=C([C@H](C)NC(=O)[C@@H]3C[C@@H](O)CN3C(=O)[C@@H](NC(=O)COCCOCCOC3=CC=C(CN4C(=O)/C(=C/C=C/C5=CC=C([N+](=O)[O-])C=C5)C5=CC=CC=C54)C=C3)C(C)(C)C)C=C2)SC=N1	No	21
**PRODB_1574**	α-syn	O=C1CCC(N2C(=O)C3=CC=C(N4CCN(CCCO[C@H]5C[C@H](OC6=CC=C7NC8=CC=C([N+](=O)[O-])C=C8SC7=C6)C5)CC4)C=C3C2=O)C(=O)N1	No	21
**PRODB_1575**	α-syn	CC1=C(C2=CC=C([C@H](C)NC(=O)[C@@H]3C[C@@H](O)CN3C(=O)[C@@H](NC(=O)CN3CCN(CCN4CCC(O[C@H]5C[C@H](OC6=CC=C7NC8=CC=C([N+](=O)[O-])C=C8SC7=C6)C5)CC4)CC3)C(C)(C)C)C=C2)SC=N1	No	21
**PROTAC 1**	GSK-3β	O=C(CN1C=C(C2=C(C3=CC=C(Cl)C=C3Cl)C(=O)NC2=O)C2=CC=CC=C21)NCCCOCCCCOCCCNC1=CC=CC2=C1C(=O)N(C1CCC(=O)NC1=O)C2=O	No	22
**PT-65**	GSK-3β	NC1=NC=C(C2=CC=C(S(=O)(=O)N3CCN(CCCCC4=CN(CCOCCOCCOCCOCCOCCNC5=CC=CC6=C5C(=O)N(C5CCC(=O)NC5=O)C6=O)N=N4)CC3)C=C2)N=C1C(=O)NC1=CC=CN=C1	No	23
**QC-01-175**	Tau	O=C(CCC1=CC=C2C(=C1)[NH]C1=CC=NC=C12)NCCOCCOCCNC1=CC=CC2=C1C(=O)N(C1CCC(=O)NC1=O)C2=O	No	24
**T3**	α-syn, Tau	CC(C=C1)=CC(S2)=C1N=C2C(C=C3)=CC=C3NCCOCCOCCOCCNC(COC4=C5C(N(C(C5=CC=C4)=O)C6CCC(NC6=O)=O)=O)=O	Yes	25
**XL01126**	LRRK2	O=C([C@H]1N(C([C@@H](NC(C2(F)CC2)=O)C(C)(SC[C@@H]3CC[C@@H](CN4CCN(C(C5=CC=C(NC6=NC=C(Cl)C(NC)=N6)C(OC)=C5)=O)CC4)CC3)C)=O)C[C@H](O)C1)NCC7=CC=C(C8=C(C)N=CS8)C=C7	Yes	26
**NX-5948**	BTK	NC(C1=C(N=C(N2C[C@H](N3C(N(CC3)C)=O)CCC2)C=N1)NC4=CC=C(C5CCN(CC5)CC6CCN(C7=CN=C(C(N[C@@H]8C(NC(CC8)=O)=O)=O)C=C7)CC6)C=C4)=O	Yes	27
**CFT1946**	BRAF (V600E)	CN1C(C(C(N(CCC2=O)C(N2)=O)=N1)=C3)=CC(N(CC4)CCC4(O)CC(N5CCC6(C[C@@H](N(C=NC7=CC=C8OC(C(F)=CC=C9N[S](N(C)CC)(=O)=O)=C9C#N)C(C7=C8)=O)CO6)CC5)=O)=C3F	Yes	28
**ARV-102**	LRRK2	CN1C(=O)N(C2CCC(=O)NC2=O)C2=C1C=C(C=C2)N1CCC(CC1)O[C@H]1CC[C@H](CN2CCN(CC2)C2=CC(=NC=N2)C2=NNC3=C2C=C(OC2(C)CC2)N=C3)CC1	Yes	NA
**20**	RET	CCC1(CCN(CC1)C1=NC=C(C=C1)C1=NC(=CN2N=CC(C#N)=C12)C1=CN(N=C1)[C@H]1CC[C@@H](CC1)N1CCC(CC1)C1=CC=C(NC2CCC(=O)NC2=O)C=C1F)C(=O)NC(C)C	Yes	29

<u>Table S4</u>. *Full data related to the collected 30 PROTACs.* *Detailed data and/or publications for two degraders undergoing clinical trial (ARV-102, NX-5948) were either partially available or missing, therefore the resulting information is fragmentary. As regards in vivo data, C. elegans (employed in 2/23 studies) was not considered.*

Information		Value	Details
Literature search	**Collected studies**	23	-
	**Collected PROTACs**	30	-
	**Research timespan**	July 2017 – October 2025	-
	**Studies without full publication**	2/23	ARV-102, NX-5948
	**PROTACs with fragmentary information available**	2/30	
BBB/cell permeability evaluation	**PROTACs tested in *in vitro* BBB models**	3/30 (10%)	PROTAC 1 and PT-65 (PAMPA-BBB), T3 (Transwell co-culture)
	**PROTACs tested via other membrane permeability assays**	2/30 (7%)	XL01126 (Caco-2), NX-5948 (MDCK-MDR1)
Resources for cell-based assays	**Cell lines used for *in vitro* assays (all instances, see Table S5)**	~ 60 (7 clusters)	Human Immortalized (65%)
			Human Primary (7%)
			Human iPSC-derived (5%)
			Mouse Immortalized (12%)
			Mouse Primary (7%)
			Rat Immortalized (3%)
			Dog Immortalized (2%)
	**Cell lines' provenance**	CNS (32%)	-
		Non-CNS (65%)	
		Neuronal-like, non-CNS (3%)	
In vivo studies	**Studies using animal models | Total instances**	11/23 (48%) | 20	Mouse (70%), Rat (20%), NHP (10%)
	**Animal models' clusters**	3	
	**PROTACs tested in vivo**	11/30 (37%)	C004019, C8, I3, KH1, PT-65, T3, XL01126, NX-5948, CFT1946, ARV-102, 20
	**Routes of Administration | Total instances**	5 | 17	In CNS (12%), Intravenous (24%), Intraperitoneal (12%), Oral (47%), Subcutaneous (6%)
Relevance	**PROTACs reported as BBB-permeable (i.e., quantified in the brain)**	9/30 (30%)	C004019, I3, KH1, T3, XL01126, NX-5948, CFT1946, ARV-102, 20
	**PROTACs in clinical trials**	3/30 (10%)	ARV-102, CFT1946, NX-5948

Table S5 (separate Excel file). *Cell lines that were used and reported in the papers for in vitro compound characterization. Each article was separately inspected and all employed cell lines used for in vitro assays were annotated. Out of 22 available articles, 60 cell lines were identified overall (Table S3). Importantly, these 60 instances were not unique, meaning that the same cell line was used in different publications. Each cell model was then annotated with information on origin and type, presence in the CNS (3 clusters: CNS, non-CNS, neuronal non-CNS), then classified based on the species (human, mouse, rat, etc) and cell type (primary, iPSC-derived, immortalized), for a total count of 7 distinct clusters. A comprehensive cluster was finally assigned to each instance, and the frequency of each class was ultimately plotted. TableS5.xlsx provides the full annotation.*

<u>Table S6</u>. *PROTACs in clinical trials with their target and SMILES codes.* *This set was last updated in September 2025. The following table also reports the 3 degraders in CT already presented in Table S3 (ARV-102, CFT1946, NX-5948).*

PROTAC name	Target	SMILES
**ARV-102**	LRRK2	CN1C(=O)N(C2CCC(=O)NC2=O)C2=C1C=C(C=C2)N1CCC(CC1)O[C@H]1CC[C@H](CN2CCN(CC2)C2=CC(=NC=N2)C2=NNC3=C2C=C(OC2(C)CC2)N=C3)CC1
**ARV-110**	AR	O=C(C1=NN=C(N2CCC(CN3CCN(C4=CC5=C(C(N(C(CC6)C(NC6=O)=O)C5=O)=O)C=C4F)CC3)CC2)C=C1)N[C@H]7CC[C@H](OC8=CC=C(C#N)C(Cl)=C8)CC7
**ARV-393**	ER	ClC1=CN=C(N2CCC(O[C@@H]3C[C@@H](N4CCC(C5=C(F)C(CN(C6CCC(NC6=O)=O)C7=O)=C7C=C5)CC4)C3)CC2)N=C1NC8=CC(C=C(OCC(NC)=O)C(N9C(C)C)=O)=C9C=C8
**ARV-471**	ER	O=C([C@@H](N(CC1=C2C=CC(N3CCN(CC4CCN(C5=CC=C([C@H]6[C@@H](C7=CC=CC=C7)CCC8=C6C=CC(O)=C8)C=C5)CC4)CC3)=C1)C2=O)CC9)NC9=O
**ARV-766**	AR	CC(C)([C@@H](C1(C)C)NC(C2=CC=C(N3CCC(CC3)CN4CCN(C5=CC(F)=C(C=C5)C(N[C@@H]6C(NC(CC6)=O)=O)=O)CC4)C=C2)=O)[C@@H]1OC7=CC(OC)=C(C=C7)C#N
**ASP-3082**	KRAS G12D	CC(C(C(C(C1CC1)=CC(C(N2[C@@](CN3)([H])C[C@@]3([H])C2)=N4)=C5N=C4OC6CCOCC6)=C5OCC7=CC=C(C(N=N8)=CN8[C@@H](C(C)C)C(N(C[C@@H]9O)[C@@H](C9)C(N[C@H](C%10=CC=C(C(SC=N%11)=C%11C)C=C%10)CO)=O)=O)C=C7)=C(C=NN%12)C%12=C%13)=C%13F
**BGB-16673**	BTK	CC1=C([C@@H](C)NC(C2=NC(C(C)(C)C)=NO2)=O)C=CC(C3=C4C(NC(C5=CN=C(N6CCN(CC6)CC7CCN(C8=CC=C(N9C(NC(CC9)=O)=O)C=C8)CC7)C=C5)=C4)=NC=N3)=C1
**BMS-986458**	BCL6	C[C@@H]1CN(CC[C@H]1NC2=CC=C3C(N(N=C3C4CCC(NC4=O)=O)C)=C2)C5=NC=C(C(NC6=CC7=C(N(C(C7)=O)C)C=C6)=N5)Cl
**CC-94676 (BMS-986365)**	AR	N#CC1=C(C(F)(F)F)C=C(N2C(N(C3=CC=C(OCCN4C[C@@H](C)N(CC(NC5=CC=CC(NC6C(NC(CC6)=O)=O)=C5)=O)CC4)C(CC)=C3)C(C)(C)C2=O)=S)C=C1
**CFT1946**	BRAF (V600E)	CN1C(C(C(N(CCC2=O)C(N2)=O)=N1)=C3)=CC(N(CC4)CCC4(O)CC(N5CCC6(C[C@@H](N(C=NC7=CC=C8OC(C(F)=CC=C9N[S](N(C)CC)(=O)=O)=C9C#N)C(C7=C8)=O)CO6)CC5)=O)=C3F
**CFT8634**	BRD9	CC(C(C1=CC(OC)=C(C(OC)=C1)CN2CC(F)([C@@H](N3CCN(C4=C(C=C(C=C4)N[C@@H]5C(NC(CC5)=O)=O)F)CC3)CC2)F)=CN6C)=C(C)C6=O
**DT-2216**	BCL-XL	C[C@H](NC(=O)[C@@H]1C[C@@H](O)CN1C(=O)[C@@H](NC(=O)CCCCCC(=O)N1CCN(CC[C@H](CSC2=CC=CC=C2)NC2=C(C=C(C=C2)S(=O)(=O)NC(=O)C2=CC=C(C=C2)N2CCN(CC3=C(CCC(C)(C)C3)C3=CC=C(Cl)C=C3)CC2)S(=O)(=O)C(F)(F)F)CC1)C(C)(C)C)C1=CC=C(C=C1)C1=C(C)N=CS1
**FHD-609**	BRD9	COC1=C(CN2CCC(CN3CCC4(CN(C5=CC(C(N([C@@H]6C(NC(CC6)=O)=O)C7)=O)=C7C=C5)C4)CC3)CC2)C(OC)=CC(C(C8=C9C=NC(N%10CCC%10)=C8)=CN(C)C9=O)=C1
**KT-253**	MDM2	ClC1=CC2=C([C@]3(C4(CCCCC4)N[C@@H](C(N[C@H]5CC[C@H](C(N6CCC(C7=CC=C8C(N(C)C(N8C9CCC(NC9=O)=O)=O)=C7)CC6)=O)CC5)=O)[C@@H]3C%10=C(F)C(Cl)=CC=C%10)C(N2)=O)C=C1
**KT-333**	STAT3	O=C([C@H]1N(C([C@H](C2)NC(C3=CC4=CC(C([P](O)(O)=O)=O)=CC=C4N3)=O)=O)[C@](CCN2C(C)=O)([H])CC1)N[C@@H](CCC(N)=O)COC(C=CC=C5CCCC(N[C@@H](C(C)(C)C)C(N(C[C@@H]6O)[C@@H](C6)C(N[C@H](C7=CC=C(C(SC=N8)=C8C)C=C7)C)=O)=O)=O)=C5Cl
**KT-413**	IRAK4	O=C(N1C2C(NC(CC2)=O)=O)C3=CC=CC(NCCC(C4)CC4(C5)CN5C[C@H]6CC[C@H](C(SC7=C8)=NC7=CC(C(C)(O)C)=C8NC(C9=CC=CC(C(F)(F)F)=N9)=O)CC6)=C3C1=O
**KT-474**	IRAK4	O=C1N(C(CCC2=O)C(N2)=O)C3=CC=CC(C#CCOC(CC4)CCN4C[C@H]5CC[C@@](N(N=C6C(F)F)C=C6NC(C7=C(N=C(N8[C@@H](CO9)C[C@@H]9C8)C=C%10)N%10N=C7)=O)([H])CC5)=C3N1C
**NX-2127**	BTK, IKZF1/3	O=C1C2=CC(N3C[C@H](CN4CCC(C5=CC=C(C=C5)NC6=C(N=CC(N7CCCCC7)=N6)C(N)=O)CC4)CC3)=CC=C2C(N1C8C(NC(CC8)=O)=O)=O
**NX-5948**	BTK	NC(C1=C(N=C(N2C[C@H](N3C(N(CC3)C)=O)CCC2)C=N1)NC4=CC=C(C5CCN(CC5)CC6CCN(C7=CN=C(C(N[C@@H]8C(NC(CC8)=O)=O)=O)C=C7)CC6)C=C4)=O
**PRT3789**	SMARCA2	CC1=C(SC=N1)C2=CC=C(C=C2)[C@@H](NC([C@@H]3C[C@H](CN3C([C@H](C(C)C)C4=CC(O[C@@H](C)CN5CC[C@H](C5)N6CCN7C8=C(NC[C@H]7C6)N=NC(C9=C(C=CC=C9)O)=C8)=NO4)=O)O)=O)C
**RNK-05047**	BRD4	CC(C)C1=CC(C2=NN=C(O)N2C2=CC=C(CN3CCN(CC4CCN(CC4)C(=O)C[C@@H]4N=C(C5=C(SC(C)=C5C)N5C(C)=NN=C45)C4=CC=C(Cl)C=C4)CC3)C=C2)=C(O)C=C1O

3 Methods

Principal component analysis

The Principal Component Analysis (PCA) was performed with DataWarrior *v*06.05.02 (https://openmolecules.org/datawarrior/index.html)[30] with default settings and by using 3 components. Variance explained by the PC1, PC2 and PC3 was 80.35%, 8.43% and 5.77%, respectively.

Calculation of 2D physicochemical descriptors

All the properties used in this study were calculated starting from the SMILES codes available in **Tables S3** and **S6**. Molecular weight (MW), number of carbon atoms (nC), Kier's flexibility index (PHI), number of hydrogen bond donors and acceptors (HDon, nHAcc), topological polar surface area (TPSA) and number of rotatable bonds (nRotB) were calculated using alvaDesc *v*1.0.18, n 2020 (http://www.alvascience.com/alvadesc/),[31] while the cLogP was obtained through DataWarrior. Strongest acidic and basic pKa values for the 9 BBB-permeable PROTACs were retrieved from the online Calculators Playground by Chemaxon (https://playground.calculators.cxn.io/).

Additional references

(1) He, M.; Cao, C.; Ni, Z.; Liu, Y.; Song, P.; Hao, S.; He, Y.; Sun, X.; Rao, Y. PROTACs: Great Opportunities for Academia and Industry (an Update from 2020 to 2021). *Signal Transduct. Target. Ther.* **2022**, *7* (1).

(2) Fang, Y.; Wang, J.; Zhao, M.; Zheng, Q.; Ren, C.; Wang, Y.; Zhang, J. Progress and Challenges in Targeted Protein Degradation for Neurodegenerative Disease Therapy. *J. Med. Chem.* **2022**, *65* (17), 11454–11477.

(3) Bertran-Mostazo, A.; Putriūtė, G.; Álvarez-Berbel, I.; Busquets, M. A.; Galdeano, C.; Espargaró, A.; Sabate, R. Proximity-Induced Pharmacology for Amyloid-Related Diseases. *Cells* **2024**, *13* (5).

(4) Chaulagain, B.; Gothwal, A.; Lamptey, R. N. L.; Trivedi, R.; Mahanta, A. K.; Layek, B.; Singh, J.; Chaulagain, B.; Gothwal, A.; Lamptey, R. N. L.; Trivedi, R.; Mahanta, A. K.; Layek, B.; Singh, J. Experimental Models of In Vitro Blood–Brain Barrier for CNS Drug Delivery: An Evolutionary Perspective. *International Journal of Molecular Sciences 2023, Vol. 24,* **2023**, *24* (3).

(5) Canfield, S. G.; Stebbins, M. J.; Morales, B. S.; Asai, S. W.; Vatine, G. D.; Svendsen, C. N.; Palecek, S. P.; Shusta, E. V. An Isogenic Blood-Brain Barrier Model Comprising Brain Endothelial Cells, Astrocytes, and Neurons Derived from Human Induced Pluripotent Stem Cells. *J. Neurochem.* **2017**, *140* (6), 874–888.

(6) Blanchard, J. W.; Bula, M.; Davila-Velderrain, J.; Akay, L. A.; Zhu, L.; Frank, A.; Victor, M. B.; Bonner, J. M.; Mathys, H.; Lin, Y. T.; Ko, T.; Bennett, D. A.; Cam, H. P.; Kellis, M.; Tsai, L. H. Reconstruction of the Human Blood-Brain Barrier in Vitro Reveals a Pathogenic Mechanism of APOE4 in Pericytes. *Nat. Med.* **2020**, *26* (6), 952–963.

(7) Adams, S. M.; Sharp, M. G. F.; Walker, R. A.; Brammar, W. J.; Varley, J. M. Differential Expression of Translation-Associated Genes in Benign and Malignant Human Breast Tumours. *Br. J. Cancer* **1992**, *65* (1), 65–71.

(8) Tomoshige, S.; Nomura, S.; Ohgane, K.; Hashimoto, Y.; Ishikawa, M. Discovery of Small Molecules That Induce the Degradation of Huntingtin. *Angewandte Chemie - International Edition* **2017**, *56* (38), 11530–11533.

(9) Wen, T.; Chen, J.; Zhang, W.; Pang, J. Design, Synthesis and Biological Evaluation of α-Synuclein Proteolysis-Targeting Chimeras. *Molecules* **2023**, *28* (11).

(10) Iida, T.; Itoh, Y.; Takahashi, Y.; Miyake, Y.; Zamani, F.; Yamashita, Y.; Takada, Y.; Akiyama, T.; Ibaraki, J.; Okuda, K.; Tokuda, Y.; Nishimura, T.; Hidaka, K.; Mori, H.; Oba, M.; Suzuki, T. Identification of Proteolysis Targeting Chimeras (PROTACs) for Lysine Demethylase 5 and Their Neurite Outgrowth-Promoting Activity. *Chem. Pharm. Bull. (Tokyo).* **2024**, *72* (7), 638–647.

(11) Tong, Y.; Zhu, W.; Chen, J.; Wen, T.; Xu, F.; Pang, J. Discovery of Small-Molecule Degraders for Alpha-Synuclein Aggregates. *J. Med. Chem.* **2023**, *66* (12), 7926–7942.

(12) Pedrini, M.; Iannielli, A.; Meneghelli, L.; Passarella, D.; Broccoli, V.; Seneci, P. Synthesis and Preliminary Characterization of Putative Anle138b-Centered PROTACs against α-Synuclein Aggregation. *Pharmaceutics* **2023**, *15* (5).

(13) Wang, W.; Zhou, Q.; Jiang, T.; Li, S.; Ye, J.; Zheng, J.; Wang, X.; Liu, Y.; Deng, M.; Ke, D.; Wang, Q.; Wang, Y.; Wang, J. Z. A Novel Small-Molecule PROTAC Selectively Promotes Tau Clearance to Improve Cognitive Functions in Alzheimer-like Models. *Theranostics* **2021**, *11* (11), 5279–5295.

(14) Yao, D.; Li, T.; Yu, L.; Hu, M.; He, Y.; Zhang, R.; Wu, J.; Li, S.; Kuang, W.; Yang, X.; Liu, G.; Xie, Y. Selective Degradation of Hyperphosphorylated Tau by Proteolysis-Targeting Chimeras Ameliorates Cognitive Function in Alzheimer's Disease Model Mice. *Front. Pharmacol.* **2024**, *15*.

(15) Silva, M. C.; Nandi, G.; Donovan, K. A.; Cai, Q.; Berry, B. C.; Nowak, R. P.; Fischer, E. S.; Gray, N. S.; Ferguson, F. M.; Haggarty, S. J. Discovery and Optimization of Tau Targeted Protein Degraders Enabled by Patient Induced Pluripotent Stem Cells-Derived Neuronal Models of Tauopathy. *Front. Cell. Neurosci.* **2022**, *16*.

(16) Liang, M.; Gu, L.; Zhang, H.; Min, J.; Wang, Z.; Ma, Z.; Zhang, C.; Zeng, S.; Pan, Y.; Yan, D.; Shen, Z.; Huang, W. Design, Synthesis, and Bioactivity of Novel Bifunctional Small Molecules for Alzheimer's Disease. *ACS Omega* **2022**, *7* (30), 26308–26315.

(17) Hatcher, J. M.; Zwirek, M.; Sarhan, A. R.; Vatsan, P. S.; Tonelli, F.; Alessi, D. R.; Davies, P.; Gray, N. S. Development of a Highly Potent and Selective Degrader of LRRK2. *Bioorg. Med. Chem. Lett.* **2023**, *94*.

(18) Tseng, Y. L.; Lu, P. C.; Lee, C. C.; He, R. Y.; Huang, Y. A.; Tseng, Y. C.; Cheng, T. J. R.; Huang, J. J. T.; Fang, J. M. Degradation of Neurodegenerative Disease-Associated TDP-43 Aggregates and Oligomers via a Proteolysis-Targeting Chimera. *J. Biomed. Sci.* **2023**, *30* (1).

(19) Holmqvist, A.; Kocaturk, N. M.; Duncan, C.; Riley, J.; Baginski, S.; Marsh, G.; Cresser-Brown, J.; Maple, H.; Juvonen, K.; Sathe, G.; Morrice, N.; Sutherland, C.; Read, K. D.; Farnaby, W. Discovery of a CNS Active GSK3 Degrader Using Orthogonally Reactive Linker Screening. *Nature Communications 2025 16:1* **2025**, *16* (1), 8857-.

(20) Jiang, X.; Zhou, J.; Wang, Y.; Liu, X.; Xu, K.; Xu, J.; Feng, F.; Sun, H. PROTACs Suppression of GSK-3β, a Crucial Kinase in Neurodegenerative Diseases. *Eur. J. Med. Chem.* **2021**, *210*.

(21) Kargbo, R. B. PROTAC Compounds Targeting α-Synuclein Protein for Treating Neurogenerative Disorders: Alzheimer's and Parkinson's Diseases. *ACS Med. Chem. Lett.* **2020**, *11* (6), 1086–1087.

(22) Guardigni, M.; Pruccoli, L.; Santini, A.; Simone, A. De; Bersani, M.; Spyrakis, F.; Frabetti, F.; Uliassi, E.; Andrisano, V.; Pagliarani, B.; Fernández-Gómez, P.; Palomo, V.; Bolognesi, M. L.; Tarozzi, A.; Milelli, A. PROTAC-Induced Glycogen Synthase Kinase 3β Degradation as a Potential Therapeutic Strategy for Alzheimer's Disease. *ACS Chem. Neurosci.* **2023**, *14* (11), 1963–1970.

(23) Qu, L.; Li, S.; Ji, L.; Luo, S.; Ding, M.; Yin, F.; Wang, C.; Luo, H.; Lu, D.; Liu, X.; Peng, W.; Kong, L.; Wang, X. Discovery of PT-65 as a Highly Potent and Selective Proteolysis-Targeting Chimera Degrader of GSK3 for Treating Alzheimer's Disease. *Eur. J. Med. Chem.* **2021**, *226*.

(24) Silva, M. C.; Ferguson, F. M.; Cai, Q.; Donovan, K. A.; Nandi, G.; Patnaik, D.; Zhang, T.; Huang, H. T.; Lucente, D. E.; Dickerson, B. C.; Mitchison, T. J.; Fischer, E. S.; Gray, N. S.; Haggarty, S. J. Targeted Degradation of Aberrant Tau in Frontotemporal Dementia Patient-Derived Neuronal Cell Models. *Elife* **2019**, *8*.

(25) Zhu, W.; Zhang, W.; Chen, J.; Tong, Y.; Xu, F.; Pang, J. Discovery of Effective Dual PROTAC Degraders for Neurodegenerative Disease-Associated Aggregates. *J. Med. Chem.* **2024**, *67* (5), 3448–3466.

(26) Liu, X.; Kalogeropulou, A. F.; Domingos, S.; Makukhin, N.; Nirujogi, R. S.; Singh, F.; Shpiro, N.; Saalfrank, A.; Sammler, E.; Ganley, I. G.; Moreira, R.; Alessi, D. R.; Ciulli, A. Discovery of XL01126: A Potent, Fast, Cooperative, Selective, Orally Bioavailable, and Blood-Brain Barrier Penetrant PROTAC Degrader of Leucine-Rich Repeat Kinase 2. *J. Am. Chem. Soc.* **2022**, *144* (37), 16930–16952. https://doi.org/10.1021/jacs.2c05499.

(27) Robbins, D. W.; Noviski, M.; Rountree, R.; Tan, M.; Brathaban, N.; Ingallinera, T.; Karr, D. E.; Kelly, A.; Konst, Z.; Ma, J.; Tenn-McClellan, A.; McKinnell, J.; Perez, L.; Guiducci, C.; Hansen, G.; Sands, A. Nx-5948, a Selective Degrader of BTK with Activity in Preclinical Models of Hematologic and Brain Malignancies. *Blood* **2021**, *138* (Supplement 1), 2251–2251.

(28) Kreger, B. T.; Liang, Y.; Reilly, N. M.; Spears, M. E.; Scott, W. A.; Simard, J. R.; Li, P.; Chaturvedi, P.; Agafonov, R. V; Stephenson, J.; Baddour, J.; Hart, J. A.; Bahadduri, P. M.; Riegel, L.; Cole, K.; Lobbardi, R.; Follmer, N. E.; Hurh, E.; Good, A.; Fitzgerald, M. E.; Patel, J.; Jackson, K. L.; Poling, L. L.; Phillips, A. J.; Nasveschuk, C. G.; Fisher, S. L.; Pollock, R. M.; Sowa, M. E. CFT1946 Is an Orally Available Brain-Penetrant BRAF V600-Mutant Degrader That Overcomes BRAF Inhibitor Resistance. Running Title CFT1946 Is an Oral BRAF V600-Mutant Selective Degrader.

(29) Orsi, D. L.; Lazarski, K. E.; Improgo, R.; Agafonov, R. V.; Ahn, J. Y.; Baddour, J.; Cassidy, K.; Chaturvedi, P.; Cole, K. S.; Deibler, R. W.; Elam, W. A.; Fitzgerald, M. E.; Garza, V. J.; Good, A.; Hulton, C. H.; Isasa, M.; Jackson, K. L.; Li, P.; Liang, Y.; Michael, R. E.; O'Shea, M. W.; Moustakim, M.; Perino, S.; Rahman, F.; Schnaderbeck, M. J.; Stone, N. P.; Tillotson, B.; Veits, G. K.; Vogelaar, A.; Yap, J. L.;

Yu, R. T.; Huang, H.; Henderson, J. A. Discovery of an Orally Bioavailable, CNS Active Pan-Mutant RET Kinase Heterobifunctional Degrader. *RSC Med. Chem.* **2025**.

(30) Sander, T.; Freyss, J.; Von Korff, M.; Rufener, C. DataWarrior: An Open-Source Program For Chemistry Aware Data Visualization And Analysis. *J. Chem. Inf. Model.* **2015**, *55* (2), 460–473.

(31) Mauri, A. AlvaDesc: A Tool to Calculate and Analyze Molecular Descriptors and Fingerprints. *Methods in Pharmacology and Toxicology* **2020**, 801–820.
